# Comparing the safety and efficacy of systemic therapies for high-risk biochemically recurrent hormone-sensitive prostate cancer: a network meta-analysis

**DOI:** 10.3389/fonc.2025.1638405

**Published:** 2025-08-29

**Authors:** Armen Aprikian, Andrew Chilelli, Thomas McLean, Anchen Nasr, Arijit Ganguli, Alexis Serikoff, Franco Guzman, Ifigeneia Barouma, Maria Zacharioudaki, Stephen J. Freedland

**Affiliations:** ^1^ McGill University, Montreal, QC, Canada; ^2^ Astellas Pharma Europe, Addlestone, United Kingdom; ^3^ Global Health Economics & Outcomes Research (HEOR), Astellas Pharma, IL, United States; ^4^ Global Medical Affairs, Astellas Pharma, IL, United States; ^5^ IQVIA Ltd, Athens, Greece; ^6^ IQVIA Commercial GmbH & Co OHG, Munich, Germany; ^7^ Cedars-Sinai Medical Center, Los Angeles, CA, United States; ^8^ Durham Veterans Affairs (VA) Medical Center, Durham, NC, United States

**Keywords:** biochemical recurrence, enzalutamide, hormone-sensitive, network meta-analysis, prostatic neoplasms, systematic review

## Abstract

**Introduction:**

Enzalutamide is the only androgen receptor pathway inhibitor approved by the United States Food and Drug Administration and the European Medicines Agency to treat high-risk biochemically recurrent non-metastatic hormone-sensitive prostate cancer. The objective of this network meta-analysis was to provide indirect evidence of the efficacy of enzalutamide relative to other therapies for biochemical recurrence after definitive therapy.

**Materials and Methods:**

We conducted a systematic literature review to identify trials that assessed the efficacy and safety of current and emerging interventions. Outcomes of interest were metastasis-free survival, overall survival, time to prostate-specific antigen progression, time to castration resistance, proportion of patients with prostate-specific antigen <0.2 ng/ml at 36 (± 4) weeks of treatment, and grade ≥3 treatment-related adverse events. Fixed- and random-effects models were run under the Bayesian framework.

**Results:**

Enzalutamide with androgen-deprivation therapy (i.e., combination therapy) demonstrated superiority over most comparators for overall survival (except androgen-deprivation therapy + docetaxel, which was similar), and over all comparators for metastasis-free survival, time to prostate-specific antigen progression, and time to castration resistance. Enzalutamide combination therapy demonstrated superiority over enzalutamide monotherapy for all efficacy outcomes, and similar performance for safety. Enzalutamide monotherapy demonstrated superiority over androgen-deprivation therapy alone and androgen-deprivation therapy + docetaxel for metastasis-free survival and time to prostate-specific antigen progression. Treatment-related adverse events were least common for androgen-deprivation therapy alone.

**Discussion:**

This network meta-analysis provides evidence that enzalutamide combination therapy provides considerable oncological benefit in high-risk biochemically recurrent non-metastatic hormone-sensitive prostate cancer, albeit with a higher risk of treatment-related adverse events.

## 1 Introduction

Approximately one-third of patients who receive primary localized treatment for prostate cancer experience biochemical recurrence within 10 years (1, 2). This recurrence is characterized by a rise in prostate-specific antigen (PSA) levels and is associated with a higher risk of worse clinical outcomes (2). To determine the best treatment approach for patients with biochemical recurrence, risk classification systems have been developed, as described in the American Society of Clinical Oncology Clinical Practice Guidelines (2021) (3) and prostate cancer guidelines from the European Association of Urology-European Association of Nuclear Medicine-European Society for Radiotherapy and Oncology-European Society of Urogenital Radiology-International Society of Geriatric Oncology (EAU-EANM-ESTRO-ESUR-SIOG) (4). According to these guidelines, patients are classified as having high-risk biochemical recurrence after radical prostatectomy if they have a PSA doubling time of <10–12 months or a Gleason score of 8–10, whereas high-risk biochemical recurrence after radiotherapy (RT) requires an interval from primary therapy to biochemical failure of <18 months or an initial biopsy Gleason score of 8–10 (3, 4). By contrast, guidelines from the American Urological Association/American Society for Radiation Oncology/Society of Urologic Oncology (AUA/ASTRO/SUO) (2024) define patients at high risk of developing metastasis with biochemical recurrence after local treatment as those with a PSA doubling time of <12 months, a shorter time to biochemical recurrence, or a higher grade or stage of disease (5, 6).

Before the EMBARK trial, specific treatment guidelines for patients with high-risk biochemically recurrent (BCR) non-metastatic hormone-sensitive prostate cancer (nmHSPC, also known as non-metastatic castration-sensitive prostate cancer [nmCSPC]) were limited and variable (1, 3, 7). Patients with nmHSPC have no detectable metastases according to conventional imaging, but if left untreated, many will eventually develop metastatic disease that can be seen on conventional imaging (8). While some guidelines recommend observation, others recommend continuous androgen-deprivation therapy (ADT), and yet others recommend intermittent ADT (i.e., an ADT regimen that includes periods of treatment suspension between ADT cycles in the absence of signs of biochemical or clinical progression) (9, 10). Although previous studies had demonstrated that enzalutamide in combination with ADT improved survival in patients with mHSPC (11, 12), little research had been conducted into the effects of this combination therapy in patients with nmHSPC. To address this gap, EMBARK—a phase 3, three-arm randomized trial that included 1068 patients—was conducted to evaluate the use of enzalutamide for high-risk BCR nmHSPC (13–15). The results of EMBARK demonstrated that enzalutamide with or without ADT was associated with improved oncological outcomes, with no new observed safety signals or decrease in quality of life (13, 16).

Based on the findings of EMBARK, enzalutamide is now the only androgen receptor pathway inhibitor approved by the United States Food and Drug Administration (FDA) (17) and European Medicines Agency (18) for the treatment of high-risk BCR nmHSPC, and it is specifically named as a preferred systemic treatment option for high-risk BCR nmHSPC by the National Comprehensive Cancer Network^®^ (NCCN) (10) and the European Association of Urology (9). However, other novel therapies may also have potential benefits in the treatment of BCR nmHSPC. Therefore, the objective of this network meta-analysis (NMA) was to provide indirect evidence of the relative efficacy of enzalutamide versus current and emerging systemic therapies for the management of patients with high-risk BCR nmHSPC whose disease has progressed after definitive therapy.

## 2 Materials and methods

We conducted a systematic literature review (SLR) to form the evidence base for the NMA. Searches were conducted on April 13, 2022, and October 3, 2023. Articles and conference abstracts were obtained by searching the Embase^®^, MEDLINE^®^, and Cochrane^®^ databases. Search strategies are presented in **Supplementary Tables 1A–E**. We also conducted manual searches on October 3, 2023, of conference records, trial registries, and reference lists of existing systematic literature reviews. A protocol was not prepared for this review. This review was conducted in accordance with the Preferred Reporting Items for Systematic Reviews and Meta-Analysis (PRISMA) guidelines.

### 2.1 Eligibility

Eligible trials assessed the efficacy and safety of systemic drug interventions in adult patients with high-risk BCR nmHSPC (**Table 1**). Eligible patients were adults who (a) had been diagnosed with histologically or cytologically confirmed adenocarcinoma of the prostate at initial biopsy, (b) had previously received definitive therapy, (c) had non-metastatic disease according to bone scan or computed tomography/magnetic resonance imaging assessment, (d) were ineligible for salvage RT (SRT), and (e) had increasing PSA levels with features of high-risk disease. Eligibility criteria for patients were intentionally broad to account for variability in how patients were selected across trials. Eligible interventions were those suggested for high-risk BCR nmHSPC following definitive therapy, which therefore included regimens of hormonal therapies with or without chemotherapy, and could include either continuous or intermittent ADT, as well as other pharmacological agents assessed for use in high-risk BCR nmHSPC. Interventions using SRT were excluded as SRT is generally recommended for patients with relatively low PSA levels (5), and some patients with biochemical recurrence may therefore be unsuitable for it.^3^Interventions that focused on supplements or lifestyle changes were also excluded.

**Table 1:** SLR eligibility criteria.

PICOS	Inclusion criteria	Exclusion criteria
Population of interest	Adult patients (≥18 years) with high-risk BCR nmHSPC^a^	• Non-human subjects • Patients aged <18 years • Study population not representative of high-risk BCR nmHSPC
Interventions of interest^b^	• Enzalutamide monotherapy • Enzalutamide plus ADT (including LHRH agonists— goserelin, histrelin, leuprolide, and triptorelin; LHRH antagonists—degarelix and relugolix; antiandrogen monotherapy—bicalutamide, flutamide, nilutamide, apalutamide, and darolutamide)	Publications not reporting interventions listed in the inclusion criteria
Comparators of interest	• Surgical castration o Bilateral orchiectomy • Hormonal castration o LHRH agonist—goserelin, histrelin, leuprolide, and triptorelin o LHRH antagonist—degarelix and relugolix • Antiandrogen monotherapy o Bicalutamide, flutamide, nilutamide, apalutamide, and darolutamide • Inhibitors of testosterone/androgen synthesis monotherapy/ combination therapy o Abiraterone • ADT plus antiandrogen (apalutamide and darolutamide)	• Non-hormonal therapies • Watchful waiting/active surveillance • Surgery o Radical prostatectomy regardless of surgical technique o Pelvic lymph node dissection • Therapies not approved or not yet at phase 2 or 3 settings in the nmHSPC setting • Salvage radiation therapy o External beam radiation therapy (EBRT): ▪ Stereotactic body radiation therapy ▪ Proton beam radiation therapy ▪ Three-dimensional conformal radiation therapy ▪ Intensity-modulated radiation therapy ▪ Image-guided radiation therapy o Internal radiation (brachytherapy), used alone or in combination with EBRT, ADT or both: ▪ Low–dose rate brachytherapy ▪ High–dose rate brachytherapy
Outcomes of interest	• Overall survival • Progression-free survival • Metastasis-free survival • Prostate cancer–specific survival • Time to castration resistance • Time to PSA progression • Time to first use of new antineoplastic therapy • Time to distant metastasis • Time to symptomatic progression • Time to first symptomatic skeletal event • Time to clinically relevant pain • Time to treatment discontinuation • Time to treatment resumption • Patient-reported outcomes (e.g., EORTC-QLQ-C30, FACT-P, EQ-5D-5L, EQ-5D-3L, BPI-SF, and EORTC-QLQ-PR25) • Adverse events	Publications not reporting outcomes listed in the inclusion criteria
Study design of interest	• Phase 2 and phase 3 randomized controlled trials • SLRs and meta-analyses^c^	• Preclinical and phase 1 studies • Non-randomized prospective controlled clinical trials or single-arm trials • Observational studies • Registry studies • Prognostic studies • Case reports • Reviews • Expert opinion • Commentaries • Letters
Restrictions	• Language restrictions: English language only • Geographic restriction: none • Time frame^d^: First phase: o All databases (except Embase): 2012- April 13, 2022 Second phase: o All databases: 2022-October 3, 2023 o Embase: 2012-October 3, 2023	Publications in languages other than English Publications published prior to 2012

ADT, androgen-deprivation therapy; BCR, biochemically recurrent; BFI-SF, Brief Pain Inventory – Short Form; DT, doubling time; EBRT, external beam radiation therapy; EQ-5D-5L, EuroQol 5-Dimension 5-Level Health Assessment Instrument; EQ-5D-3L, EuroQol 5-Dimension 3-Level Health Assessment Instrument; FACT-P, Functional Assessment of Cancer Therapy–Prostate; LHRH, luteinizing hormone–releasing hormone; nmHSPC, non-metastatic hormone-sensitive prostate cancer; PSA, prostate-specific antigen; RT, radiotherapy; SLR, systematic literature review; EORTC-QLQ-PR25, EORTC Quality of Life Questionnaire-Prostate 25.

a nmHSPC also referred to as non-metastatic hormone-naive prostate cancer, non-metastatic castration-sensitive prostate cancer (nmCSPC), or non-metastatic androgen-dependent prostate cancer.

b The listed intervention and comparators of interest correspond to those of interest to answer the SLR research questions. These therapies do not correspond to the intervention and the comparator of the studies to be selected.

c SLRs and NMAs were included at the abstract review stage in order to search their reference lists for any additional studies, and they were subsequently excluded during the full text review stage.

d The searches were run in two phases. In the first phase, the search was conducted on April 13, 2022, and articles from 2012 to April 13, 2022, were searched from the above listed databases except Embase. The second phase was conducted on October 3, 2023, where articles were searched from 2012 to October 3, 2023, in Embase, and for all the remaining databases, the articles were searched from 2022 to October 3, 2023.

### 2.2 Screening and data extraction

Records were screened for eligibility by two independent researchers. Disagreements were resolved by a third independent reviewer. Data were extracted by one researcher into a data extraction form, and a quality check was performed by a second independent reviewer.

### 2.3 Feasibility assessment

A feasibility assessment was conducted for each outcome of interest to determine whether an NMA was possible for the outcome. Efficacy outcomes of interest were overall survival (OS), metastasis-free survival (MFS), time to PSA progression, time to castration resistance, and the proportion of patients who achieved PSA <0.2 ng/ml at 36 (± 4) weeks of treatment. To compare time to PSA progression across studies, studies that evaluated time to PSA progression and those that covered PSA progression-free survival were both included for this outcome, as these terms are often used interchangeably. The safety endpoint of interest was the occurrence of any type of grade ≥3 treatment-related adverse events (TRAEs). The feasibility assessment included an evaluation of heterogeneity across the trials identified in the SLR (e.g., study design, population, comparators, outcome definitions, and patient baseline characteristics) and an appraisal of whether sufficient consistent data were available for each outcome of interest. Risk of bias was assessed by one researcher using the Cochrane Risk of Bias 2 (RoB-2) tool, and a quality check was performed by a second independent reviewer.

### 2.4 Statistical analyses

Statistical analyses were conducted under the Bayesian framework (19) and run with R (version 4.2.1 or higher) (20) using the “multinma” package (21). Four Markov chain Monte Carlo chains with different starting values were used for all models, with a burn-in of at least 2000 iterations and a further sample of at least 10,000 iterations. Fixed- and random-effects models were run, with the latter accounting for heterogeneity among studies. Given the small number of studies informing each treatment comparison in the networks of evidence, fixed-effects models were used for inference.

The proportional hazards assumption was evaluated for the EMBARK trial using visual inspection of log-cumulative hazard plots, the Grambsch–Therneau statistical test for the scaled Schoenfeld residuals, and visual inspection of Schoenfeld residuals. As all other trials in the NMA reported constant hazard ratios based on Cox proportional hazards models, the proportional hazards assumption was assumed to be met.

Where appropriate, sensitivity analyses were conducted to evaluate the influence of studies with a high risk of bias according to the RoB-2 tool or where the feasibility assessment identified potential sources of clinical or methodological heterogeneity. The feasibility of conducting subgroup analyses was explored but was ultimately not deemed viable due to the low number of studies reporting subgroup results.

### 2.5 Ethics statement

Ethics clearance was not required for this study as all analyses were conducted on previously published or presented data.

## 3 Results

**Figure 1:**
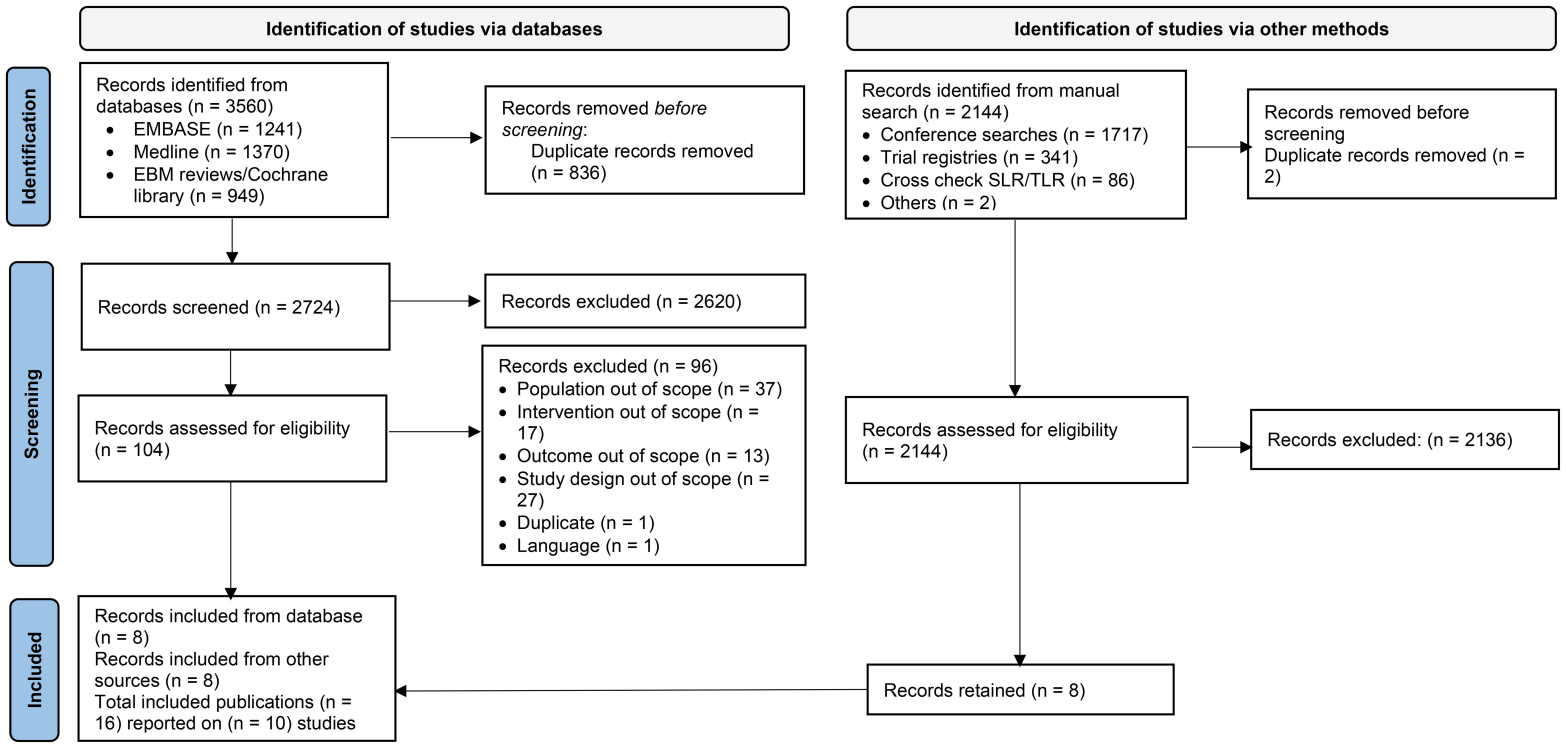
PRISMA flow diagram of study selection process. EBM, evidence-based medicine; SLR, systematic literature review; TLR, targeted literature review.

A total of 3560 citations were identified in the SLR, from which 16 publications or presentations based on 10 eligible trials were included in the NMA (**Figure 1**). The characteristics of the 10 trials are presented in **Table 2**, and trial design details are presented in **Supplementary Table 2**.

**Table 2:** Trial characteristics.

Author, year (trial name)	Arms	Age (in years); median (range)	Median follow-up period (months)	Serum PSA (ng/mL); median (range)	PSADT (in months); median (range)	Gleason score ≥8, n (%)	ECOG PS, n (%)	Definitive treatment, n (%)	Other previous treatment, n (%)
EMBARK (14, 15, 22, 23)	Enzalutamide + ADT	69 (51–87)	NR	5.0 (1.0–308.3)	4.6 (0.9–9.6)	120 (33.8)	PS0: 328 (92.4) PS1: 26 (7.3) PS2: 1 (0.3)	RP: 269 (75.8) RT: 265 (74.6) RT and RP: 179 (50.4)	ADT: 107 (30.1)
	ADT	70 (50–92)	NR	5.5 (1.1–163.3)	5.0 (1.1–10.8)	113 (31.6)	PS0: 336 (93.9) PS1: 21 (5.9) PS2: 0.0 (0.0)	RP: 254 (70.9) RT: 283 (79.1) RT and RP: 179 (50)	ADT: 113 (31.6)
	Enzalutamide	69 (49–93)	NR	5.3 (1.1–37.0)	5.0 (1.0–18.9)	111 (31.3)	PS0: 321 (90.4) PS1: 34 (9.6) PS2: 0.0 (0.0)	RP: 265 (74.6) RT: 256 (72.1) RT and RP: 166 (46.8)	ADT: 112 (31.5)
Autio, 2021 (NA) (24)	Abiraterone acetate	64 (43–84)	NR	3.1 (1.2–35.4)	NR	17 (43.6)	PS0: 35 (90.0) PS1: 4 (10.0)	RP: 39 (100.0)	SRT: 23 (59.0)
	Abiraterone acetate + ADT	65 (53–74)	NR	5.8 (1.2–45.1)	NR	19 (46.3)	PS0: 39 (95.0) PS1: 2 (5.0)	RP: 41 (100.0)	SRT: 24 (59.0)
	ADT	66 (46–78)	NR	4.1 (1.0–48.3)	NR	17 (40.5)	PS0: 39 (93.0) PS1: 3 (7.0)	RP: 42 (100.0)	SRT: 27 (64.0)
Crook, 2012 (SWOG-JPR7) (25)	Continuous ADT	74.4 (45.3–88.9)	6.9	3–15 ng/mL: 535 (76.9) >15 ng/mL: 160 (23.0)	NR	NR	PS0: 568 (81.6) PS1: 127 (18.2)	RT: 696 (100.0) RP: 79 (11.4)	ADT: 271 (38.9)
	Intermittent ADT	74.2 (29.4–89.7)	6.9	3–15 ng/mL: 531 (77.0) >15 ng/mL: 159 (23.0)	NR	NR	PS0: 548 (79.4) PS1: 142 (20.6)	RT: 690 (100.0) RP: 79 (11.4)	ADT: 271 (39.3)
Duchesne, 2016 (TOAD) (26)	Delayed ADT	70.0 (50.7–85.0)	5.0	NR	<10 months: 57 (42.0%) ≥10 months: 80 (58.0%)	NR	NR	RT: 88 (64.0) RP ± RT: 49 (36.0)	Neoadjuvant ADT: 69 (50.0)
	Immediate ADT	71.1 (54.0–88.0)	5.0	NR	<10 months: 60 (48.0%) ≥10 months: 64 (52.0%)	NR	NR	RT: 77 (62.0) RP ± RT: 47 (38.0)	Neoadjuvant ADT: 56 (45.0)
Morris, 2021 (TAX3503) (27)	ADT + docetaxel	66.0 (NR)	33.6	0.8 (0.5–1.4)	3.9 (IQR: 2.6–6.1)	61 (29.5)	NR	RP: 100 RT: 65 (31.4)	ADT ^a^: 14 (21.5)
	ADT	65.0 (NR)	33.6	0.7 (0.5–1.7)	3.9 (IQR: 2.5–3.5)	56 (27.2)	NR	RP: 100 RT: 76 (36.9)	ADT ^a^: 18 (23.7)
Oudard, 2019 (AOM 03108) (28)	ADT + docetaxel	64.0 (NR)	30.0	2.6 (1.0–6.2)	5.8 (IQR: 3.2–8.4)	41 (32.8)	PS0: 119 (95.2) PS1: 4 (3.2)	RP: 90 (72.0) RT± ADT: 35 (28.0)	SRT: 54 (43.2)
	ADT	66.0 (NR)	30.0	2.9 (1.0–6.0)	5.8 (IQR: 3.7–9.1)	36 (28.8)	PS0: 116 (92.8) PS1: 8 (6.4)	RP: 92 (73.6) RT± ADT: 33 (26.4)	SRT: 56 (44.8)
Spetsieris, 2021 (FINITE) (29, 30)	Abiraterone acetate + ADT	65.0 (44.0–80.0)	64.4	1.2 (0.2–11.1)	NR	41 (41.0)	NR	RP: 93 (94.0) RP + RT: 48 (48.0) RT: 6 (6.0)	NR
	ADT	65.0 (42.0–85.0)	64.4	1.0 (0.2–33.3)	NR	40 (40.0)	NR	RP: 93 (95.0) RP + RT: 49 (50.0) RT: 5 (5.0)	NR
Josefsson 2023 (SPCG-14) (31)	Bicalutamide + docetaxel	NR^b^	4.9	3.0 (1.7–7.4)	<6 months: 100 (63.0%) ≥6 months: 58 (37.0%)	NR^b^	NR^b^	RP: 52 (30) RP + salvage: 61 (35) RT: 44 (26)	Cryotherapy: 1 (<1) No curative: 14 (8)
	Bicalutamide	NR^b^	4.9	3.7 (1.8–8.2)	<6 months: 99 (63.0%) ≥6 months: 58 (37.0%)	NR^b^	NR^b^	RP: 45 (26) RP + salvage: 63 (36) RT: 49 (28)	Cryotherapy: 1 (<1) No curative: 17 (10)
Aggarwal 2023 (PRESTO, AFT-19) (32)	ADT	NR	NR	NR	NR	NR	NR	NR	NR
	ADT + apalutamide	NR	NR	NR	NR	NR	NR	NR	NR
	ADT + apalutamide + abiraterone acetate plus prednisone	NR	NR	NR	NR	NR	NR	NR	NR
NCT01790126 (33)	Apalutamide	66.1 (6.18)^c^	NR	NR	NR	NR	NR	NR	NR
	LHRH agonist	67.3 (6.51)^c^	NR	NR	NR	NR	NR	NR	NR
	Apalutamide + LHRH agonist	67.5 (6.67)^c^	NR	NR	NR	NR	NR	NR	NR

ADT, androgen-deprivation therapy; ECOG PS, Eastern Cooperative Oncology Group Performance Status; HT, hormone therapy; IQR, interquartile range; LHRH, luteinizing hormone–releasing hormone; NR, not reported; PSA, prostate-specific antigen; PSADT, prostate-specific antigen doubling time; RP, radical prostatectomy; RT, radiotherapy; SRT, salvage radiotherapy.

a ADT use was reported only for those who had received previous RT.

b The reported values concerned the total population of patients, irrespective of whether or not they had had prior curative treatment.

c Mean age was reported instead of median.

Some data described as “not reported” have since been published, but were not available at the time of the NMA

In terms of heterogeneity, baseline patient characteristics and duration of follow-up were generally comparable between studies (**Table 2**). Patients’ median age ranged from 64–74 years. Median serum PSA was lowest among patients in the Morris 2021 study (ADT alone: 0.7; ADT + docetaxel: 0.8). Follow-up periods ranged from 4.9 months to 64.4 months. The availability of each outcome is presented in **Supplementary Table 3**, and networks of evidence are presented in **Supplementary Figures 1A–F**. Outcome definitions were generally consistent across trials. In all trials, OS was defined as the time from randomization to death from any cause. For time to PSA progression, variations were noted in PSA progression thresholds across the included trials. Time to castration resistance was defined as “three increases in the PSA level at least 1 month apart or evidence of new clinical disease while the patient was receiving ADT and the testosterone was at castrate levels” in two studies (14, 25) and as “a rise in PSA while on ADT” in one study (26). For the proportion of patients with PSA < 0.2 ng/ml, EMBARK assessed serum PSA at 36 weeks (14, 15, 22, 23), while Autio et al. (2021) assessed serum PSA at 32 weeks (24). Finally, safety events were graded according to the National Cancer Institute Common Terminology Criteria for Adverse Events (CTCAE) versions 3.0 (34), 4.0 (35), or 4.03, with the exception of the SWOG-JPR7 study, where the methods for assessing adverse events (AEs) were not specified. For the purposes of this NMA, AEs in the trial by Morris et al. (27) that were not specified as “treatment emergent” but were presented by treatment arm were assumed to be treatment related, as no “unexpected” grade ≥3 AEs were reported.

In the evaluation of the proportional hazards assumption for the EMBARK trial, no pattern was identified in the Schoenfeld residual plot over time, indicating no evidence against the null hypothesis of proportional hazards for OS and MFS. Similarly, no violations of the proportional hazards assumption were identified for time to PSA progression or time to castration resistance. The results of the proportional hazards evaluation are presented in **Supplementary Figure 2**.

The results of the risk-of-bias assessment are presented in **Supplementary Table 4**. Most studies were deemed to be low risk or to have some concerns.

### 3.1 Efficacy results

Overall results for the relative efficacy of enzalutamide compared with other treatments using the fixed-effects model are presented in **Tables 3A** and **3B**. Results of the random-effects model are presented in **Supplementary Tables 5** and **6**.

**Table 3A:** League table for outcomes of interest presenting the relative efficacy of enzalutamide (monotherapy and combination therapy) versus comparator treatments.

MFS (HR, 95% CI)														
			Treatments (reference)											
			Enzalutamide			Enzalutamide + ADT				Docetaxel + ADT			ADT	
Comparators	Enzalutamide		–			1.49 (1.27–1.74)^a^				0.61 (0.38–0.98)^a^			0.63 (0.46–0.87)^a^	
	Enzalutamide + ADT		0.67 (0.57–0.79)^a^			–				0.41 (0.25–0.67)^a^			0.42 (0.30–0.61)^a^	
	Docetaxel + ADT		1.63 (1.02–2.60)^a^			2.43 (1.49–3.97)^a^				–			1.03 (0.74–1.44)	
	ADT		1.58 (1.15–2.19)^a^			2.36 (1.64–3.38)^a^				0.97 (0.70–1.35)			–	
OS (HR, 95% CI)														
			Treatments (reference)											
			Enzalutamide			Enzalutamide + ADT				Docetaxel + ADT			ADT	
Comparators	Enzalutamide		–			1.32 (1.12–1.56)^a^				1.02 (0.59–1.77)			0.78 (0.52–1.16)	
	Enzalutamide + ADT		0.76 (0.64–0.89)^a^			–				0.77 (0.43–1.37)			0.59 (0.38–0.91)^a^	
	Docetaxel + ADT		0.98 (0.57–1.70)			1.30 (0.73–2.30)				–			0.77 (0.53–1.11)	
	ADT		1.28 (0.86–1.91)			1.70 (1.09–2.61)^a^				1.31 (0.90–1.90)			–	
Time to PSA Progression (HR, 95% CI)														
		Treatments (reference)												
		Enzalutamide		Enzalutamide + ADT			Docetaxel + ADT		Abiraterone		Abiraterone + ADT			ADT
Comparators	Enzalutamide	–		4.85 (2.62–8.98)^a^			0.40 (0.26–0.62)^a^		0.17 (0.09–0.32)^a^		0.60 (0.39–0.93)^a^			0.33 (0.23–0.49)^a^
	Enzalutamide + ADT	0.21 (0.11–0.38)^a^		–			0.08 (0.04–0.18)^a^		0.04 (0.01–0.09)^a^		0.12 (0.06–0.26)^a^			0.07 (0.03–0.14)^a^
	Docetaxel + ADT	2.48 (1.62–3.79)^a^		12.00 (5.66–25.67)^a^			–		0.43 (0.25–0.72)^a^		1.49 (1.14–1.95)^a^			0.82 (0.68–0.99)^a^
	Abiraterone	5.81 (3.12–10.91)^a^		28.13 (11.68–68.21)^a^			2.35 (1.39–3.97)^a^		–		3.50 (2.08–5.94)^a^			1.93 (1.19–3.15)^a^
	Abiraterone + ADT	1.66 (1.08–2.55)^a^		8.05 (3.80–17.28)^a^			0.67 (0.51–0.88)^a^		0.29 (0.17–0.48)^a^		–			0.55 (0.46–0.67)^a^
	ADT	3.01 (2.05–4.43)^a^		14.57 (7.07–30.34)^a^			1.21 (1.01–1.47)^a^		0.52 (0.32–0.84)^a^		1.81 (1.50–2.19)^a^			–
Time to castration resistance (HR, 95% CI)														
					Treatments (reference)									
					Enzalutamide + ADT			Intermittent ADT				ADT		
Comparators	Enzalutamide + ADT				-			0.09 (0.05–0.16)^a^				0.07 (0.04–0.13)^a^		
	Intermittent ADT				11.00 (6.33–19.14)^a^			–				0.80 (0.66–0.97)^a^		
	ADT				13.73 (7.63–24.66)^a^			1.25 (1.03–1.51)^a^				–		

ADT, androgen-deprivation therapy; CI, confidence interval; HR, hazard ratio; MFS, metastasis-free survival; OS, overall survival; PSA, prostate-specific antigen.

a Statistically significant.

**Table 3B:** League table for outcomes of interest presenting the relative efficacy of enzalutamide (monotherapy and combination therapy) versus comparator treatments.

Undetectable PSA (OR, 95% CI)						
		Treatment (reference)				
		Enzalutamide	Enzalutamide + ADT	ADT	Abiraterone	Abiraterone + ADT
Comparators	Enzalutamide	–	0.25 (0.11–0.51)^a^	3.72 (2.45–5.77)^a^	1.40 (0.39–4.41)	0.98 (0.26–3.22)
	Enzalutamide + ADT	3.99 (1.9–9.02)^a^	–	14.82 (7.68–31.98)^a^	5.59 (1.43–20.37)^a^	3.90 (0.95–14.85)
	ADT	0.27 (0.17–0.41)^a^	0.07 (0.0–0.13)^a^	–	0.37 (0.12–1.10)	0.26 (0.08–0.79)^a^
	Abiraterone	0.72 (0.23–2.56)	0.18 (0.05–0.70)^a^	2.67 (0.91–8.63)	–	0.70 (0.18–2.63)
	Abiraterone + ADT	1.02 (0.31–3.83)	0.26 (0.07–1.05)	3.79 (1.26–13.15)^a^	1.43 (0.38–5.45)	–
Grade ≥3 TEAEs (OR, 95% CI)						
		Treatments (reference)						
		Enzalutamide + ADT	ADT	Enzalutamide	Docetaxel + ADT	
Comparators	Enzalutamide + ADT	–	2.24 (1.42–3.57)^a^	1.11 (0.75–1.66)	0.29 (0.14–0.58)^a^	
	ADT	0.45 (0.28–0.71)^a^	–	0.50 (0.31–0.79)^a^	0.13 (0.08–0.22)^a^	
	Enzalutamide	0.90 (0.60–1.33)	2.01 (1.27–3.25)^a^	–	0.26 (0.13–0.53)^a^	
	Docetaxel + ADT	3.44 (1.73–6.93)^a^	7.71 (4.64–13.33)^a^	3.83 (1.89–7.75)^a^	–	

ADT, androgen-deprivation therapy; CI, confidence interval; PSA, prostate-specific antigen; TRAE, treatment-related adverse event.

a Statistically significant.

#### 3.1.1 Overall survival

**Figure 2:**
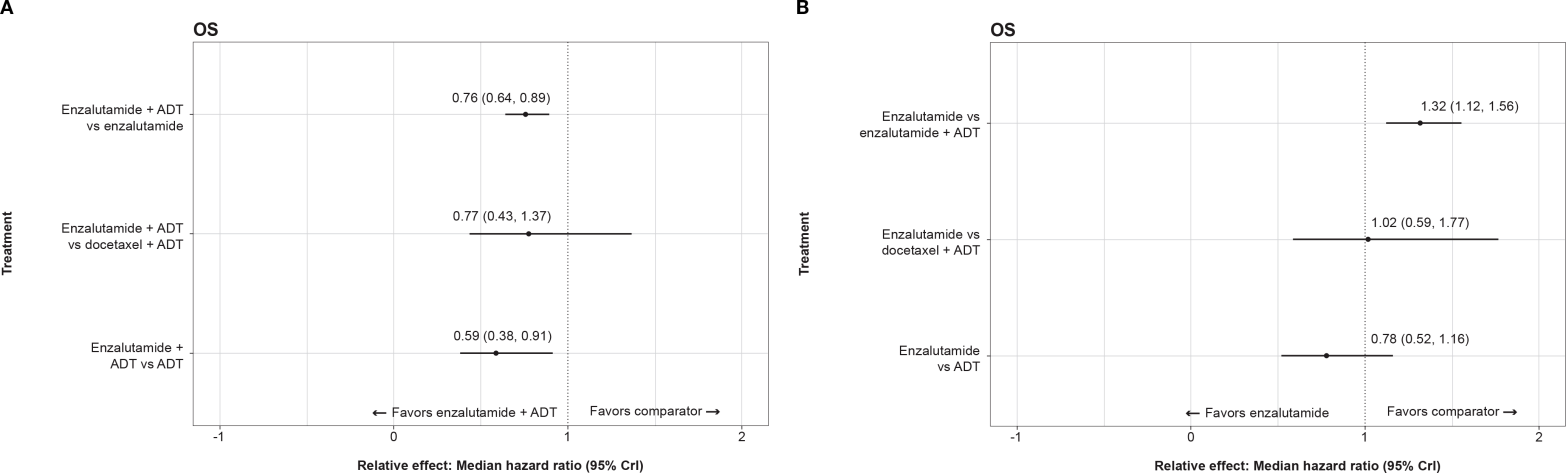
**(A)** Forest plot representing the relative efficacy of enzalutamide combined with ADT against other active treatments in the analysis of overall survival. **(B)**. Forest plot representing the relative efficacy of enzalutamide monotherapy against other active treatments in the analysis of overall survival. ADT, androgen-deprivation therapy; CrI, credible interval; OS, overall survival.

For OS, enzalutamide combination therapy (i.e., enzalutamide with ADT) demonstrated superiority over ADT alone and enzalutamide monotherapy (i.e., ADT alone) but similar performance to ADT + docetaxel (**Figure 2A**). Enzalutamide monotherapy demonstrated similar performance to ADT + docetaxel and ADT alone (**Figure 2B**).

#### 3.1.2 Metastasis-free survival

For MFS, enzalutamide combination therapy demonstrated superiority over all comparators. Specifically, both enzalutamide combination therapy and enzalutamide monotherapy demonstrated superiority over ADT + docetaxel and ADT alone (**Supplementary Figures 3A, B**). Enzalutamide combination therapy demonstrated superiority over enzalutamide monotherapy.

#### 3.1.3 Time to prostate-specific antigen progression

For PSA progression, enzalutamide combination therapy demonstrated superiority over all comparators (**Supplementary Figure 3C**). Enzalutamide monotherapy also demonstrated superiority over most comparators (ADT + docetaxel, ADT + abiraterone, abiraterone monotherapy, and ADT alone), but was inferior to enzalutamide combination therapy (**Supplementary Figure 3D**).

#### 3.1.4 Time to castration resistance

For time to castration resistance, enzalutamide combination therapy demonstrated superiority over ADT alone (**Supplementary Figure 3E**).

#### 3.1.5 Prostate-specific antigen < 0.2 ng/ml at 36 (± 4) weeks of treatment

For the proportion of patients with PSA < 0.2 ng/ml at 36 (± 4) weeks of treatment, enzalutamide combination therapy demonstrated superiority over abiraterone monotherapy and ADT alone (**Supplementary Figure 3F**). Although the results for enzalutamide combination therapy were numerically favorable compared with ADT + abiraterone, they did not indicate superiority. Enzalutamide monotherapy demonstrated inferiority compared to enzalutamide combination therapy; however, it demonstrated superiority over ADT alone and similar performance to ADT + abiraterone and abiraterone monotherapy (**Supplementary Figure 3G**).

### 3.2 Safety (grade ≥3 TRAEs)

In terms of safety, enzalutamide combination therapy demonstrated superiority over ADT + docetaxel and inferiority compared with ADT alone (**Supplementary Figure 3H**). Enzalutamide monotherapy demonstrated superiority over ADT + docetaxel and inferiority to ADT alone (**Supplementary Figure 3I**). Enzalutamide combination therapy demonstrated similar performance to enzalutamide monotherapy.

### 3.3 Sensitivity analyses

Sensitivity analyses were conducted to assess the impact of studies that had a high risk of bias or heterogeneity, which could affect the results of the base case analysis. Four such studies were identified: first, in Duchesne et al. (2016) (TOAD) (26), more than 50% of patients had PSA doubling time ≥10 months, and only around 40% of patients in each arm were classified as having high-risk disease. Second, Morris et al. (2021) (TAX3503) (27) included patients with relatively low serum PSA levels at baseline, suggesting a much earlier stage of biochemical recurrence compared with patients in other trials. Third, Spetsieris et al. (2021) (FINITE) (29) provided limited information regarding baseline patient characteristics, and median serum PSA levels in their study were relatively lower than those reported in other trials. Finally, Aggarwal 2023 (PRESTO, AFT-19) (32) was an ongoing study with only preliminary results available. The results of three sensitivity analyses conducted to evaluate the impact of excluding Duchesne et al. (2016) (26), Morris et al. (2021) (27), and Spetsieris et al. (2021) (29), as well as one conducted on the impact of including Aggarwal et al. (2023) (32), did not differ from the results of the base case analysis. Details are presented in **Supplementary Tables 7A, B**.

## 4 Discussion

Patients with high-risk BCR have several different treatment options. To systematically compare treatments, we conducted, to our knowledge, the first NMA focusing on the effectiveness of enzalutamide for the treatment of high-risk BCR nmHSPC. Our analysis included 16 citations based on 10 phase 2/3 trials. We assessed the efficacy of multiple treatments on several clinically important endpoints, including OS, MFS, and time to PSA progression. Finally, to explore the impact of trial heterogeneity on our conclusions, we conducted a wide range of sensitivity analyses and found consistent results across all scenarios. We found that enzalutamide combination therapy demonstrated superiority over ADT alone and enzalutamide monotherapy for OS and over all comparators for MFS (vs enzalutamide monotherapy, ADT alone, and ADT + docetaxel) and time to castration resistance (vs ADT alone). It also demonstrated superiority to all comparators for time to PSA progression (vs enzalutamide monotherapy, abiraterone monotherapy, ADT + abiraterone, ADT + docetaxel, and ADT alone, as well as ADT + apalutamide and ADT + abiraterone + apalutamide in sensitivity analysis). Importantly, for oncological outcomes, no treatment was superior to enzalutamide combination therapy. Likewise, enzalutamide monotherapy demonstrated superiority to ADT alone and ADT + docetaxel in terms of MFS and time to PSA progression. These benefits must be balanced against the higher rates of grade ≥3 TRAEs with enzalutamide (with or without ADT) relative to other options except ADT + docetaxel, which was associated with more grade ≥3 TRAEs than enzalutamide. Together, the findings support the use of enzalutamide with or without ADT as a new standard of care for patients with high-risk BCR.

The benefits identified for enzalutamide in this analysis are aligned with treatment recommendations already included in current clinical guidelines. Specifically, the NCCN guidelines list enzalutamide as a preferred treatment option for patients with high-risk BCR nmHSPC (10). Similarly, the 2024 EAU guidelines contain a “strong” recommendation for providers to offer enzalutamide with or without ADT to patients with high-risk BCR nmHSPC (9). Further, our analysis is consistent with previous safety signals (36). In terms of the rate of grade ≥3 TRAEs, enzalutamide with and without ADT demonstrated superiority over ADT + docetaxel. The greater toxicity of docetaxel identified in our analysis, combined with a lack of greater clinical benefit, supports current recommendations for the use of enzalutamide in this patient population. Moreover, recent evidence from a Canadian study suggests that, compared with ADT alone, ADT + enzalutamide is cost-effective at established willingness to pay thresholds, making it a preferred treatment option for patients with high-risk BCR nmHSPC (37).

This study has some limitations. Survival data from several of the trials included in the NMA, including the EMBARK trial, are relatively immature, as the median survival time had not been reached for all survival outcomes at the time of data cut-off for each study; this could potentially lead to implausible estimates of survival benefit. However, previous trials that reported immature OS data for non-metastatic castration-resistant prostate cancer (nmCRPC) (PROSPER, SPARTAN, and ARAMIS) and metastatic hormone-sensitive prostate cancer (mHSPC) (ARCHES and TITAN) found that data maturity strengthened the results, and eventually, statistically significant and clinically meaningful benefits were achieved (38–42). In other indications for the use of enzalutamide in prostate cancer (nmCRPC, mHSPC, and mCRPC), a consistent effect was observed for enzalutamide at all evaluated disease stages, lending credibility to our findings (11, 12, 43–45). Within the trials included in this NMA, EMBARK had the longest follow-up period of all eligible trials that reported OS and included data from over 1000 patients. Of 271 OS events required across the treatment groups in EMBARK to achieve the protocol-defined power needed for this outcome, nearly half (n = 130, 48%) had occurred by the cut-off date, which supports these potential survival benefits.

Although both fixed- and random-effects models were run, the latter were limited by the small number of studies informing each treatment comparison in our analysis, resulting in less precise estimates. Thus, the fixed-effects models were used for inference, which does not account for between-study heterogeneity, and may, therefore, underestimate uncertainty in effect estimates As potential sources of clinical heterogeneity were identified in the evidence base, a series of sensitivity analyses were conducted to explore the impact of removing studies that deviated from the other studies in terms of baseline characteristics. After assessing the impact of excluding these studies, the overall results remained consistent, which lends credibility to our findings. In addition, the sparse evidence networks in this NMA mean that credible intervals were wide, leading to high uncertainty, particularly for the proportion of patients with undetectable PSA at 36 weeks and the rate of grade ≥3 TRAEs. Specifically, treatment-emergent and unspecified grade ≥3 AEs in the Morris study (27) were assumed to be TRAEs, which may have resulted in overestimation. However, as no unexpected grade ≥3 AEs were reported in the included trial, we do not expect this assumption to have substantially impacted the results. Additionally, treatment comparisons for TRAEs were not adjusted for time on treatment, which may have impacted our findings. Finally, we did not assess the exact make-up of TRAEs. For example, although enzalutamide combination showed superiority over enzalutamide monotherapy in multiple oncological outcomes with similar safety, there are differences in the side effect profiles of the two that may favor use of one regimen over another (i.e., better preserved sexual function with monotherapy). As such, the choice to use a particular regimen should be based on shared decision-making after evaluating efficacy, grade ≥3 TRAEs, and any specific side effects.

We evaluated the feasibility of conducting subgroup analyses, but the small number of studies reporting subgroup results meant that this was not viable. However, based on the results of the studies that conducted and reported the results of subgroup analyses (e.g., EMBARK), no groups were identified that would not be expected to benefit from treatment with enzalutamide (13).

Finally, a limitation of meta-analyses in general is that the results of more recent studies that were published or analyzed after the initial literature review was conducted will not be captured. In this study, available data on apalutamide was preliminary, or included only in sensitivity analyses, and was only available for time to PSA progression. Although enzalutamide with and without ADT demonstrated superiority over apalutamide in a sensitivity analysis, future analyses should incorporate more recently available apalutamide data to confirm this finding. Similarly, although the SLR search strategy followed best practice guidelines, there remains a risk that not all relevant studies were captured.

## 5 Conclusion

Overall, this novel NMA provides up-to-date evidence on the relative efficacy and safety profile of interventions for the treatment of high-risk BCR nmHSPC, demonstrating that enzalutamide with or without ADT provides considerable oncological benefit in high-risk BCR nmHSPC, albeit with a higher risk of TRAEs compared to ADT alone. Future research should prioritize updating this NMA to incorporate more mature OS data, as well as more recent data from the treatments evaluated in the NMA. Further research is also needed to identify predictive biomarkers that may help to identify tumors that are more sensitive to treatment with ARPIs compared with other mechanisms of action (e.g., chemotherapy).

## Data Availability

All data generated or analyzed during this study, which support the findings of this study, are included within this article and its supplementary information files. Researchers may access analysis not present in the manuscript from the corresponding author upon reasonable request. For the Astellas criteria on data sharing, see: https://www.clinicaltrials.astellas.com/transparency/.

## References

[1] Shore ND Moul JW Pienta KJ Czernin J King MT Freedland SJ . Biochemical recurrence in patients with prostate cancer after primary definitive therapy: treatment based on risk stratification. Prostate Cancer Prostatic Dis. (2024) 27:192–201. doi: 10.1038/s41391-023-00712-z, PMID: 37679602 PMC11096125

[2] Simon NI Parker C Hope TA Paller CJ . Best approaches and updates for prostate cancer biochemical recurrence. Am Soc Clin Oncol Educ Book. (2022) 42:1–8. doi: 10.1200/EDBK_351033, PMID: 35503984 PMC9844546

[3] Virgo KS Rumble RB de Wit R Mendelson DS Smith TJ Taplin ME . Initial management of noncastrate advanced, recurrent, or metastatic prostate cancer: ASCO guideline update. J Clin Oncol. (2021) 39:1274–305. doi: 10.1200/JCO.20.03256, PMID: 33497248

[4] Mottet N van den Bergh RCN Briers E Van den Broeck T Cumberbatch MG De Santis M . EAU-EANM-ESTRO-ESUR-SIOG Guidelines on Prostate Cancer-2020 update. Part 1: Screening, diagnosis, and local treatment with curative intent. Eur Urol. (2021) 79:243–62. doi: 10.1016/j.eururo.2020.09.042, PMID: 33172724

[5] Morgan TM Boorjian SA Buyyounouski MK Chapin BF Chen DYT Cheng HH . Salvage therapy for prostate cancer: AUA/ASTRO/SUO guideline part I: Introduction and treatment decision-making at the time of suspected biochemical recurrence after radical prostatectomy. J Urol. (2024) 211:509–17. doi: 10.1097/JU.0000000000003892, PMID: 38421253

[6] Lowrance W Dreicer R Jarrard DF Scarpato KR Kim SK Kirkby E . Updates to advanced prostate cancer: AUA/SUO guideline (2023). J Urol. (2023) 209:1082–90. doi: 10.1097/JU.0000000000003452, PMID: 37096583

[7] Parker C Castro E Fizazi K Heidenreich A Ost P Procopio G . Prostate cancer: ESMO Clinical Practice Guidelines for diagnosis, treatment and follow-up. Ann Oncol. (2020) 31:1119–34. doi: 10.1016/j.annonc.2020.06.011, PMID: 32593798

[8] Saad F Bogemann M Suzuki K Shore N . Treatment of nonmetastatic castration-resistant prostate cancer: focus on second-generation androgen receptor inhibitors. Prostate Cancer Prostatic Dis. (2021) 24:323–34. doi: 10.1038/s41391-020-00310-3, PMID: 33558665 PMC8134049

[9] Cornford P Tilki D van den Bergh R . EAU - EANM - ESTRO - ESUR - ISUP - SIOG Guidelines on Prostate Cancer (2024). EAU-EANM-ESTRO-ESUR-ISUP-SIOG-Guidelines-on-Prostate-Cancer-2024_2024-04-09-132035_ypmy_2024-04-16-122605_lqpk.pdf.

[10] Referenced with permission from the NCCN Clinical Practice Guidelines in Oncology (NCCN Guidelines®) for Prostate Cancer V.2.2025. © National Comprehensive Cancer Network, Inc . To view the most recent and complete version of the guideline, go online to NCCN.org. NCCN makes no warranties of any kind whatsoever regarding their content, use or application and disclaims any responsibility for their application or use in any way (202).

[11] Armstrong AJ Azad AA Iguchi T Szmulewitz RZ Petrylak DP Holzbeierlein J . Improved survival with enzalutamide in patients with metastatic hormone-sensitive prostate cancer. J Clin Oncol. (2022) 40:1616–22. doi: 10.1200/JCO.22.00193, PMID: 35420921 PMC9113211

[12] Davis ID Martin AJ Stockler MR Begbie S Chi KN Chowdhury S . Enzalutamide with standard first-line therapy in metastatic prostate cancer. N Engl J Med. (2019) 381:121–31. doi: 10.1056/NEJMoa1903835, PMID: 31157964

[13] Freedland SJ de Almeida Luz M De Giorgi U Gleave M Gotto GT Pieczonka CM . Improved outcomes with enzalutamide in biochemically recurrent prostate cancer. N Engl J Med. (2023) 389:1453–65. doi: 10.1056/NEJMoa2303974, PMID: 37851874

[14] Freedland SJ Gleave ME De Giorgi U Rannikko A Pieczonka C Sridharan S . 1778P Treatment (tx) of high-risk biochemically recurrent prostate cancer with enzalutamide (enza) in combination with leuprolide acetate (LA): Secondary endpoints from EMBARK. Annal Oncol. (2023) 34:S961. doi: 10.1016/j.annonc.2023.09.2728

[15] De Giorgi U Freedland SJ Gleave ME Tutrone R Bailen JL Roos E . 1777P Enzalutamide (enza) monotherapy for the treatment (tx) of prostate cancer with high-risk biochemical recurrence (BCR): EMBARK secondary endpoints. Annal Oncol. (2023) 34:S960–1. doi: 10.1016/j.annonc.2023.09.2727

[16] Freedland SJ Gleave M De Giorgi U Rannikko A Pieczonka CM Tutrone RF . Enzalutamide and quality of life in biochemically recurrent prostate cancer. NEJM Evid. (2023) 2:EVIDoa2300251. doi: 10.1056/EVIDoa2300251, PMID: 38320501

[17] United States Food and Drug Administration . FDA approves enzalutamide for non-metastatic castration-sensitive prostate cancer with biochemical recurrence (2023). Available online at: https://www.fda.gov/drugs/resources-information-approved-drugs/fda-approves-enzalutamide-non-metastatic-castration-sensitive-prostate-cancer-biochemical-recurrence (Accessed 14 January 2025).

[18] European Medicines Agency . Xtandi (enzalutamide) (2024). Available online at: https://www.ema.europa.eu/en/medicines/human/EPAR/xtandiproduct-info (Accessed 14 January 2025).

[19] Dias S Welton NJ Sutton AJ Ades AE . NICE DSU Technical Support Document 2: A generalised linear modelling framework for pairwise and network meta-analysis of randomised controlled trials. London: NICE Decision Support Unit Technical Support Documents (2014). 27466657

[20] R Core Team . R: A language and environment for statistical computing. MSOR Connections (2014). p. 1.

[21] Phillippo DM . multinma: Bayesian network meta-analysis of individual and aggregate data (2020). Available online at: https://dmphillippo.github.io/multinma/ (Accessed 14 January 2025).

[22] Freedland S Gleave M De Giorgi U Rannikko A Pieczonka C Tutrone R . 1766MO Health-related quality of life (HRQoL) in nonmetastatic hormone-sensitive prostate cancer (nmHSPC) patients (pts) with high-risk biochemical recurrence (BCR) from the EMBARK study. Annal Oncol. (2023) 34:S955. doi: 10.1016/j.annonc.2023.09.2716

[23] Shore ND de Almeida Luz M De Giorgi U Gleave M Gotto GT Haas GP . LBA02–09 EMBARK: A phase 3 randomized study of enzalutamide or placebo plus leuprolide acetate and enzalutamide monotherapy in high-risk biochemically recurrent prostate cancer. J Urol. (2023) 209:e1190. doi: 10.1097/JU.0000000000003361.09 37119051

[24] Autio KA Antonarakis ES Mayer TM Shevrin DH Stein MN Vaishampayan UN . Randomized phase 2 trial of abiraterone acetate plus prednisone, degarelix, or the combination in men with biochemically recurrent prostate cancer after radical prostatectomy. Eur Urol Open Sci. (2021) 34:70–8. doi: 10.1016/j.euros.2021.09.015, PMID: 34934969 PMC8655386

[25] Crook JM O’Callaghan CJ Duncan G Dearnaley DP Higano CS Horwitz EM . Intermittent androgen suppression for rising PSA level after radiotherapy. N Engl J Med. (2012) 367:895–903. doi: 10.1056/NEJMoa1201546, PMID: 22931259 PMC3521033

[26] Duchesne GM Woo HH Bassett JK Bowe SJ D'Este C Frydenberg M . Timing of androgen-deprivation therapy in patients with prostate cancer with a rising PSA (TROG 03.06 and VCOG PR 01–03 [TOAD]): A randomised, multicentre, non-blinded, phase 3 trial. Lancet Oncol. (2016) 17:727–37. doi: 10.1016/S1470-2045(16)00107-8, PMID: 27155740

[27] Morris MJ Mota JM Lacuna K Hilden P Gleave M Carducci MA . Phase 3 randomized controlled trial of androgen deprivation therapy with or without docetaxel in high-risk biochemically recurrent prostate cancer after surgery (TAX3503). Eur Urol Oncol. (2021) 4:543–52. doi: 10.1016/j.euo.2021.04.008, PMID: 34020931 PMC9386576

[28] Oudard S Latorzeff I Caty A Migliancio L Sevin E Hardy-Bessard AC . Effect of adding docetaxel to androgen-deprivation therapy in patients with high-risk prostate cancer with rising prostate-specific antigen levels after primary local therapy: A randomized clinical trial. JAMA Oncol. (2019) 5:623–32. doi: 10.1001/jamaoncol.2018.6607, PMID: 30703190 PMC6512307

[29] Spetsieris N Boukovala M Alafis I Davis J Zurita A Wang X Tu SM . Abiraterone acetate plus prednisone in non-metastatic biochemically recurrent castration-naïve prostate cancer. Eur J Cancer. (2021) 157:259–67. doi: 10.1016/j.ejca.2021.06.017, PMID: 34536949

[30] Hahn AW Wang X Efstathiou E Hwang H Zurita AJ Spetsieris N . Third analysis of a randomized trial of finite abiraterone acetate (AA) plus LHRH agonist (LHRHa) versus LHRHa in biochemically recurrent, non-metastatic hormone-naïve prostate cancer (M0HNPC). J Clin Oncol. (2022) 40:135. doi: 10.1200/JCO.2022.40.6_suppl.135

[31] Josefsson A Jellvert Å Holmberg E Brasso K Meidahl Petersen P Aaltomaa S . Effect of docetaxel added to bicalutamide in Hormone-Naïve non-metastatic prostate cancer with rising PSA, a randomized clinical trial (SPCG-14). Acta Oncologica. (2023) 62:372–80. doi: 10.1080/0284186X.2023.2199940, PMID: 37073813

[32] Aggarwal RR Heller G Hillman DW Xiao H Picus J Taplin ME . Baseline characteristics associated with PSA progression-free survival in patients (pts) with high-risk biochemically relapsed prostate cancer: Results from the phase 3 PRESTO study (AFT-19). J Clin Oncol. (2023) 41:208. doi: 10.1200/JCO.2023.41.6_suppl.208

[33] ClinicalTrials.gov. NCT01790126 . The role of highly selective androgen receptor (AR) targeted therapy in men with biochemically relapsed hormone sensitive prostate cancer (2020). Available online at: https://classic.clinicaltrials.gov/ct2/show/NCT01790126 (Accessed 14 January 2025).

[34] Trotti A Colevas AD Setser A Rusch V Jaques D Budach V . CTCAE v3. 0: Development of a comprehensive grading system for the adverse effects of cancer treatment. Semin Radiat Oncol. (2003) 13:176–81. doi: 10.1016/S1053-4296(03)00031-6, PMID: 12903007

[35] National Cancer Institute . Common Terminology Criteria for Adverse Events. CTCAE (2009). Available online at: http://ctep.cancer.gov/protocolDevelopment/electronic_applications/ctc.htm.

[36] Saad F Hamilou Z Lattouf JB . A drug safety evaluation of enzalutamide to treat advanced prostate cancer. Expert Opin Drug Saf. (2021) 20:741–9. doi: 10.1080/14740338.2021.1919620, PMID: 34114527

[37] Aprikian A Saad F Wywial E McLean T Johnston K Li Y . Cost-effectiveness of enzalutamide with androgen-deprivation therapy (ADT) versus ADT alone for the treatment of high-risk biochemically recurrent non-metastatic castration-sensitive prostate cancer in Canada. J Med Econ. (2025) 28:766–77. doi: 10.1080/13696998.2025.2503660, PMID: 40395149

[38] Hussain M Fizazi K Saad F Rathenborg P Shore N Ferreira U . Enzalutamide in men with nonmetastatic, castration-resistant prostate cancer. N Engl J Med. (2018) 378:2465–74. doi: 10.1056/NEJMoa1800536, PMID: 29949494 PMC8288034

[39] Armstrong AJ Szmulewitz RZ Petrylak DP Holzbeierlein J Villers A Azad A . ARCHES: A randomized, phase iii study of androgen deprivation therapy with enzalutamide or placebo in men with metastatic hormone-sensitive prostate cancer. J Clin Oncol. (2019) 37:2974–86. doi: 10.1200/JCO.19.00799, PMID: 31329516 PMC6839905

[40] Saad F Cella D Basch E Hadaschik BA Mainwaring PN Oudard S . Effect of apalutamide on health-related quality of life in patients with non-metastatic castration-resistant prostate cancer: An analysis of the SPARTAN randomised, placebo-controlled, phase 3 trial. Lancet Oncol. (2018) 19:1404–16. doi: 10.1016/S1470-2045(18)30456-X, PMID: 30213449

[41] Fizazi K Shore N Tammela TL Ulys A Vjaters E Polyakov S . Nonmetastatic, castration-resistant prostate cancer and survival with darolutamide. N Engl J Med. (2020) 383:1040–9. doi: 10.1056/NEJMoa2001342, PMID: 32905676

[42] Chi KN Agarwal N Bjartell A Chung BH Pereira de Santana Gomes AJ Given R . Apalutamide for metastatic, castration-sensitive prostate cancer. N Engl J Med. (2019) 381:13–24. doi: 10.1056/NEJMoa1903307, PMID: 31150574

[43] Sternberg CN Fizazi K Saad F Shore ND De Giorgi U Penson DF . Enzalutamide and survival in nonmetastatic, castration-resistant prostate cancer. N Engl J Med. (2020) 382:2197–206. doi: 10.1056/NEJMoa2003892, PMID: 32469184

[44] Beer TM Armstrong AJ Rathkopf D Loriot Y Sternberg CN Higano CS . Enzalutamide in men with chemotherapy-naive metastatic castration-resistant prostate cancer: Extended analysis of the phase 3 PREVAIL study. Eur Urol. (2017) 71:151–4. doi: 10.1016/j.eururo.2016.07.032, PMID: 27477525 PMC5570461

[45] Merseburger AS Haas GP von Klot CA . An update on enzalutamide in the treatment of prostate cancer. Ther Adv Urol. (2015) 7:9–21. doi: 10.1177/1756287214555336, PMID: 25642291 PMC4294802

