# Supplementary material for: Comparing the safety and efficacy of systemic therapies for high-risk biochemically recurrent hormone-sensitive prostate cancer: a network meta-analysis
Source: Front Oncol. 2025 Aug 29;15:1638405. doi: 10.3389/fonc.2025.1638405 (PMC12428788; doi:10.3389/fonc.2025.1638405)
Supplement: Supplementary file 1 [file DataSheet1.docx]

**Supplementary Materials**

**eTable 1A.** **Search strategy for nmHSPC clinical reviews. Ovid MEDLINE®, epub ahead of print, in process, in data review, and other non-indexed citations; daily and Versions®; 1946 to April 12, 2022. Search conducted on April 13, 2022.**

| **ID** | **Search string** | **# Hits** |
| --- | --- | --- |
| 1 | exp Prostatic Neoplasms/ | 141,313 |
| 2 | (prostat$ adj6 (neoplas$ or canc$ or carcinoma$ or adeno$ or tumour$ or tumor$ or malignan$ or sarcoma$ or lymphoma$ or leiomyosarcoma$)).mp. | 192,737 |
| 3 | 1 or 2 | 192,737 |
| 4 | exp Neoplasms, Hormone-Dependent/ | 5910 |
| 5 | ((hormon$ or androgen or castration$) adj2 (dependen$ or naiv$ or sensitiv$)).mp. | 26,944 |
| 6 | (HSPC or HSPCa or ADPC or ADPCA or CSPC).mp. | 2118 |
| 7 | Or/4-6 | 28,893 |
| 8 | 3 and 7 | 8317 |
| 9 | (earl$ or localised or localized or locally advance$ or non metasta$ or nonmetasta$ or not-metasta$ or M0 or stage$ one or stage$ 1 or stage$ I or stage$ two or stage$ 2 or stage$ II or stage$ 2A or stage$ IIA or stage$ 2B or stage$ IIB stage$ 2C or stage$ IIC or stage$ three or stage$ 3 or stage$ III or stage$ 3A or stage$ IIIA or stage$ 3B or IIIB stage$ 3C or stage$ IIIC or stage$ four or stage$ 4 or stage$ IV or stage$ 4A or stage$ IVA).mp. | 2,466,391 |
| 10 | 8 and 9 | 1412 |
| 11 | (high$ risk$ adj10 (earl$ or localised or localized or locally advance$ or non metasta$ or nonmetasta$ or not-metasta$ or M0 or stage$ one or stage$ 1 or stage$ I or stage$ two or stage$ 2 or stage$ II or stage$ 2A or stage$ IIA or stage$ 2B or stage$ IIB stage$ 2C or stage$ IIC or stage$ three or stage$ 3 or stage$ III or stage$ 3A or stage$ IIIA or stage$ 3B or IIIB stage$ 3C or stage$ IIIC or stage$ four or stage$ 4 or stage$ IV or stage$ 4A or stage$ IVA)).mp. | 21,079 |
| 12 | 3 and 11 | 1327 |
| 13 | 10 or 12 | 2651 |
| 14 | Prostate-Specific Antigen/ | 27,842 |
| 15 | (rise$ or rising or increas$ or doubl$).mp. | 6,950,637 |
| 16 | 14 and 15 | 8841 |
| 17 | ((prostat$ specific antigen$ or PSA) adj3 (rise$ or rising or increas$ or doubl$)).mp. | 5344 |
| 18 | (biochemical adj (failure or relapse)).mp. | 3049 |
| 19 | Or/16-18 | 13,176 |
| 20 | 3 and 9 and 19 | 4587 |
| 21 | 13 or 20 | 6827 |
| 22 | watchful waiting/ | 4838 |
| 23 | (Watch$ adj2 wait$).ti,ab,kw. | 4197 |
| 24 | active surveillance.ti,ab,kw. | 9119 |
| 25 | Conservative Treatment/ | 4558 |
| 26 | ((careful$ or prostat$) adj2 monitor$).ti,ab,kw. | 11,704 |
| 27 | ((defer$ or delay$ or expectant$) adj2 (treatment$ or management$)).ti,ab,kw. | 23,275 |
| 28 | ((observ$ or surveillance) adj3 prostat$).ti,ab,kw. | 3191 |
| 29 | or/22-28 | 56,760 |
| 30 | exp Prostatectomy/ | 34,479 |
| 31 | (surger$ or adenectomy or resect$ or prostatectom$ or remov$).ti,ab,kw. | 2,194,415 |
| 32 | exp Lymph Node Excision/ | 52,393 |
| 33 | lymph node dissection.ti,ab,kw. | 20,463 |
| 34 | (PLND or RRP or RPP).ti,ab,kw. | 4157 |
| 35 | or/30-34 | 2,232,667 |
| 36 | exp Radiotherapy/ | 200,880 |
| 37 | (external beam adj3 (radiation$ or irradiation$ or RT or therap$)).ti,ab,kw. | 8593 |
| 38 | EBRT.ti,ab,kw. | 3659 |
| 39 | ((stereotactic or stereotaxic) adj3 (radiation$ or radiotherap$)).ti,ab,kw. | 11,041 |
| 40 | (SABR or SABRT or SBRT).ti,ab,kw. | 6168 |
| 41 | exp Proton Therapy/ | 4705 |
| 42 | (proton beam adj3 (radiation$ or therap$)).ti,ab,kw. | 1634 |
| 43 | PBRT.ti,ab,kw. | 165 |
| 44 | exp Radiotherapy, Conformal/ | 19,570 |
| 45 | (three dimensional conformal adj3 (radiation therap$ or radiotherap$)).ti,ab,kw. | 1776 |
| 46 | 3d-CRT.ti,ab,kw. | 1305 |
| 47 | exp Radiotherapy, Intensity-Modulated/ | 12,306 |
| 48 | (intensity modulated adj3 (therap$ or radiotherap$)).ti,ab,kw. | 11,863 |
| 49 | (IGRT or IMRT).ti,ab,kw. | 11,951 |
| 50 | exp Radiotherapy, Image-Guided/ | 3713 |
| 51 | (image guided adj3 (radiation$ therap$ or radiotherap$)).ti,ab,kw. | 2758 |
| 52 | exp Brachytherapy/ | 21,075 |
| 53 | brachytherap$.ti,ab,kw. | 19,466 |
| 54 | or/36-53 | 216,884 |
| 55 | ((surger$ or surgical) adj3 castrat$).ti,ab,kw. | 1486 |
| 56 | exp Orchiectomy/ | 15,306 |
| 57 | (orchectom$ or orcheotom$ or orchidectom$ or testectom$).ti,ab,kw. | 3430 |
| 58 | or/55-57 | 18,072 |
| 59 | exp Antineoplastic Agents, Hormonal/ | 180,882 |
| 60 | (androgen deprivation therap$ or ADT$).mp. | 10,019 |
| 61 | exp Gonadotropin-Releasing Hormone/ | 33,524 |
| 62 | (lutein$ hormon$ releas$ or LHRH$ or LH RH$).mp. | 15,024 |
| 63 | goserelin/ | 1643 |
| 64 | (goserelin or ici 118 630 or ici 118630 or ici118630 or novimp or prozoladex or reseligo or zoladex or 65807-02-5).mp. | 2000 |
| 65 | (histrelin or orf 17070 or orf17070 or "rl 0903" or rl0903 or rwj 17070 or rwj17070 or spd 424 or spd424 or supprelin or vantas or "vp 002" or vp002 or 220810-26-4).mp. | 135 |
| 66 | leuprolide/ | 3015 |
| 67 | (leuprorelin$ or a 43818 or a43818 or abbott 43818 or camcevi or carcinil or ckd 841 or ckd841 or daronda or depo lupron or eligard or eliprogel or elityran or enanton or enantone or fensolvi or ginecrin or klebrocid or la 2575 or la2575 or leptoprol or lerin or leuplin or leupro-sandoz or leuprogel or leuprolid or leuprolide$ or leupron or leuprone or leuprostin or lorelin or lucrin or lupride or luprolex or lupron or lutrate or ovarest or politrate or procrin or prostap or prostaplant or reliser or sixantone or sot 375 or sot375 or tap 144 or tap144 or tol 2506 or tol2506 or trenantone or viadur or vp 4896 or vp4896 or 53714-56-0 or 74381-53-6).mp. | 3820 |
| 68 | Triptorelin Pamoate/ | 1980 |
| 69 | (triptorelin$ or arvekap or ay 25650 or ay25650 or bim 21003 or bim21003 or bn 52014 or bn52014 or cl 118532 or cl118532 or debio 8200 or debio 8206 or debio8200 or debio8206 or decapeptyl or detryptorelin or diphereline or fertipeptil or gonapeptyl or isr 48 or isr48 or microrelin or moapar or ovugel or pamorelin or salvacyl or spherotide or trelstar or triptodur or wy 424222 or wy42422 or 57773-63-4).mp. | 2322 |
| 70 | (degarelix or fe 200486 or fe200486 or firmagon or 214766-78-6).mp. | 284 |
| 71 | (relugolix or mvt 601 or mvt601 or orgovyx or relumina or rvt 601 or rvt601 or t 1331285 or t1331285 or tak 385 or tak385 or 737789-87-6).mp. | 65 |
| 72 | exp Androgen Antagonists/ | 17,514 |
| 73 | (androgen antagonist$ or anti androgen$ or antiandrogen$).mp. | 17,611 |
| 74 | (bicalutamide or biluron or casodex or cosudex or ici 176334 or lutamidal or probic or raffolutil or 90357-06-5).mp. | 2019 |
| 75 | Flutamide/ | 2697 |
| 76 | (flutamide or apimid or cytamid or drogenil or etaconil or euflex or eulexin or eulexine or flucinom or fludinom or flugerel or fluken or flulem or flumid or flutamex or flutamin or flutan or flutaplex or flutax or flutol or fluxus or fugerel or niftolid or niftolide or niphtholid or odyne or prostamid or prostica or prostogenat or sch 13521 or sch13521 or sebatrol or tafenil or testac or 13311-84-7).mp. | 3847 |
| 77 | (anadron or anandron or canandron or nilandron or nitulamide or rn 23908 or ru 23908 or 63612-50-0).mp. | 98 |
| 78 | (enzalutamide or mdv 3100 or mdv3100 or xtandi or 915087-33-1).mp. | 2710 |
| 79 | (apalutamide or arn 509 or arn509 or erleada or 956104-40-8).mp. | 366 |
| 80 | (darolutamide or bay 1841788 or bay1841788 or nubeqa or odm 201 or odm201 or 1297538 32 9).mp. | 176 |
| 81 | or/59-80 | 234,871 |
| 82 | 29 or 35 or 54 or 58 or 81 | 2,665,047 |
| 83 | exp Randomized Controlled Trials as Topic/ | 158,074 |
| 84 | Double-Blind Method/ | 171,118 |
| 85 | Single-Blind Method/ | 31,804 |
| 86 | clinical trial/ | 534,634 |
| 87 | randomized controlled trial/ | 564,284 |
| 88 | pragmatic clinical trial.pt. | 2079 |
| 89 | randomized controlled trial.pt. | 564,284 |
| 90 | Random Allocation/ | 106,825 |
| 91 | placebos/ | 35,917 |
| 92 | ((singl$ or doubl$ or treb$ or tripl$) adj (blind$3 or dumm$3 or mask$3)).ti,ab,kw. | 187,687 |
| 93 | trial.ti. | 260,051 |
| 94 | placebo$.ti,ab,kw. | 235,416 |
| 95 | (randomi?ed controlled trial$ or rct).ti,ab,kw. | 232,688 |
| 96 | randomi?ation.ti,ab,kw. | 44,705 |
| 97 | (crossover or cross over).ti,ab,kw. | 93,032 |
| 98 | (random$ adj2 allocat$).ti,ab,kw. | 40,188 |
| 99 | or/83-98 | 1,406,596 |
| 100 | 21 and 82 and 99 | 873 |
| 101 | Case-Control Studies/ | 320,061 |
| 102 | Longitudinal Studies/ | 156,917 |
| 103 | Retrospective Studies/ | 1,014,465 |
| 104 | Cohort Studies/ | 310,148 |
| 105 | (cohort adj2 stud$).ti,ab,kw. | 292,483 |
| 106 | (Case control adj2 stud$).ti,ab,kw. | 122,458 |
| 107 | Observational Study/ | 124,920 |
| 108 | (observational adj2 stud$).ti,ab,kw. | 165,006 |
| 109 | Cross-Sectional Studies/ | 419,669 |
| 110 | (cross sectional adj2 stud$).ti,ab,kw. | 250,717 |
| 111 | Follow-Up Studies/ | 684,094 |
| 112 | (follow up adj2 stud$).ti,ab,kw. | 65,316 |
| 113 | exp Electronic Health Records/ | 25,527 |
| 114 | exp Medical Records/ | 155,591 |
| 115 | ((health or medical) adj3 (record$ or review$)).ti,ab,kw. | 191,045 |
| 116 | (chart adj2 (review$ or study or studies)).ti,ab,kw. | 49,633 |
| 117 | (real world adj3 (evidence or data$)).ti,ab,kw. | 12,603 |
| 118 | (database$ or data base$ or registr$).ti,ab,kw. | 862,479 |
| 119 | exp registries/ | 112,046 |
| 120 | or/101-119 | 3,685,649 |
| 121 | 21 and 82 and 120 | 1955 |
| 122 | 100 or 121 | 2516 |
| 123 | animals/ not humans/ | 4,955,959 |
| 124 | 122 not 123 | 2516 |
| 125 | Letter/ | 1,175,796 |
| 126 | editorial/ | 601,308 |
| 127 | Historical Article/ | 368,179 |
| 128 | Comment/ | 958,521 |
| 129 | Case Reports/ | 2,261,544 |
| 130 | (letter or comment$).ti. | 175,677 |
| 131 | (case reports or historical article or letter or comment or editorial).pt. | 4,431,587 |
| 132 | or/125-131 | 4,498,583 |
| 133 | 124 not 132 | 2480 |
| 134 | limit 133 to English language | 2367 |
| 135 | limit 134 to yr="2012 -Current" | 1310 |

**eTable 1B. Search strategy for nmHSPC clinical reviews. Ovid MEDLINE®, epub ahead of print, in process, in data review, and other non-indexed citations; daily and Versions®; 1946 to October 2, 2023. Search conducted on October 3, 2023.**

| **ID** | **Search string** | **# Hits** |
| --- | --- | --- |
| 1 | exp Prostatic Neoplasms/ | 149,813 |
| 2 | (prostat$ adj6 (neoplas$ or canc$ or carcinoma$ or adeno$ or tumour$ or tumor$ or malignan$ or sarcoma$ or lymphoma$ or leiomyosarcoma$)).mp. | 207,226 |
| 3 | 1 or 2 | 207,226 |
| 4 | exp Neoplasms, Hormone-Dependent/ | 5913 |
| 5 | ((hormon$ or androgen or castration$) adj2 (dependen$ or naiv$ nglishtiv$)).mp. | 28,270 |
| 6 | (HSPC or HSPCa or ADPC or ADPCA or CSPC).mp. | 2465 |
| 7 | or/4-6 | 30,521 |
| 8 | 3 and 7 | 9028 |
| 9 | (earl$ nglishsed or localized or locally advance$ or non metasta$ or nonmetasta$ or not-metasta$ or M0 or stage$ one or stage$ 1 or stage$ I or stage$ two or stage$ 2 or stage$ II or stage$ 2A or stage$ IIA or stage$ 2B or stage$ IIB stage$ 2C or stage$ IIC or stage$ three or stage$ 3 or stage$ III or stage$ 3A or stage$ IIIA or stage$ 3B or IIIB stage$ 3C or stage$ IIIC or stage$ four or stage$ 4 or stage$ IV or stage$ 4A or stage$ IVA).mp. | 2,680,647 |
| 10 | 8 and 9 | 1559 |
| 11 | (high$ risk$ adj10 (earl$ nglishsed or localized or locally advance$ or non metasta$ or nonmetasta$ or not-metasta$ or M0 or stage$ one or stage$ 1 or stage$ I or stage$ two or stage$ 2 or stage$ II or stage$ 2A or stage$ IIA or stage$ 2B or stage$ IIB stage$ 2C or stage$ IIC or stage$ three or stage$ 3 or stage$ III or stage$ 3A or stage$ IIIA or stage$ 3B or IIIB stage$ 3C or stage$ IIIC or stage$ four or stage$ 4 or stage$ IV or stage$ 4A or stage$ IVA)).mp. | 24,247 |
| 12 | 3 and 11 | 1497 |
| 13 | 10 or 12 | 2954 |
| 14 | Prostate-Specific Antigen/ | 29,879 |
| 15 | (rise$ or rising nglisheas$ or doubl$).mp. | 7,604,571 |
| 16 | 14 and 15 | 9439 |
| 17 | ((prostat$ specific antigen$ or PSA) adj3 (rise$ or rising nglisheas$ or doubl$)).mp. | 5640 |
| 18 | (biochemical adj (failure or relapse)).mp. | 3235 |
| 19 | or/16-18 | 14,076 |
| 20 | 3 and 9 and 19 | 4899 |
| 21 | 13 or 20 | 7400 |
| 22 | exp Antineoplastic Agents, Hormonal/ | 185,091 |
| 23 | (androgen deprivation therap$ or ADT$).mp. | 11,491 |
| 24 | exp Gonadotropin-Releasing Hormone/ | 34,510 |
| 25 | (lutein$ hormonglisheas$ or LHRH$ or LH RH$).mp. | 15,160 |
| 26 | goserelin/ | 1655 |
| 27 | (goserelin or ici 118 630 or ici 118630 or ici118630 or novimp or prozoladex or reseligo or zoladex or 65807-02-5).mp. | 2048 |
| 28 | (histrelin or orf 17070 or orf17070 “r "rl 0”03" or rl0903 or rwj 17070 or rwj17070 or spd 424 or spd424 or supprelin or vantas “r "vp ”02" or vp002 or 220810-26-4).mp. | 140 |
| 29 | leuprolide/ | 3073 |
| 30 | (leuprorelin$ or a 43818 or a43818 or abbott 43818 or camcevi or carcinil or ckd 841 or ckd841 or daronda or denglisron or eligard or eliprogel or elityran or enanton or enantone or fensolvi or ginecrin or klebrocid or la 2575 or la2575 or leptoprol or lerin or leuplin or leupro-sandoz or leuprogel nglishelid or leuprolide$ or leupron or leuprone or leuprostin or lorelin or lucrin or lupride or luprolex nglisron or lutrate or ovarest or politrate or procrin or prostap or prostaplant or reliser or sixantone or sot 375 or sot375 or tap 144 or tap144 or tol 2506 or tol2506 or trenantone or viadur or vp 4896 or vp4896 or 53714-56-0 or 74381-53-6).mp. | 3961 |
| 31 | Triptorelin Pamoate/ | 2005 |
| 32 | (triptorelin$ or arvekap or ay 25650 or ay25650 or bim 21003 or bim21003 or bn 52014 or bn52014 or cl 118532 or cl118532 or debio 8200 or debio 8206 or debio8200 or debio8206 or decapeptyl or detryptorelin or diphereline or fertipeptil or gonapeptyl or isr 48 or isr48 or microrelin or moapar or ovugel or pamorelin or salvacyl or spherotide nglishtar or triptodur or wy 424222 or wy42422 or 57773-63-4).mp. | 2382 |
| 33 | (degarelix or fe 200486 or fe200486 or firmagon or 214766-78-6).mp. | 312 |
| 34 | (relugolix or mvt 601 or mvt601 or orgovyx nglishina or rvt 601 or rvt601 or t 1331285 or t1331285 or tak 385 or tak385 or 737789-87-6).mp. | 117 |
| 35 | exp Androgen Antagonists/ | 18,743 |
| 36 | (androgen antagonist$ or anti androgen$ or antiandrogen$).mp. | 18,995 |
| 37 | (bicalutamide or biluron or casodex or cosudex or ici 176334 or lutamidal or probic or raffolutil or 90357-06-5).mp. | 2131 |
| 38 | Flutamide/ | 2737 |
| 39 | (flutamide or apimid or cytamid or drogenil or etaconil or euflex or eulexin or eulexine or flucinom or fludinom or flugerel or fluken or flulem or flumid or flutamex or flutamin or flutan or flutaplex or flutax or flutol or fluxus or fugerel or niftolid or niftolide or niphtholid or odyne or prostamid or prostica or prostogenat or sch 13521 or sch13521 or sebatrol or tafenil or testac or 13311-84-7).mp. | 3931 |
| 40 | (anadron or anandron or canandron or nilandron or nitulamide or rn 23908 or ru 23908 or 63612-50-0).mp. | 98 |
| 41 | (enzalutamide or mdv 3100 or mdv3100 or xtandi or 915087-33-1).mp. | 3250 |
| 42 | (apalutamide or arn 509 or arn509 or erleada or 956104-40-8).mp. | 510 |
| 43 | (darolutamide or bay 1841788 or bay1841788 or nubeqa or odm 201 or odm201 or 1297538 32 9).mp. | 299 |
| 44 | or/22-43 | 242,492 |
| 45 | exp Randomized Controlled Trials as Topic/ | 168,175 |
| 46 | Double-Blind Method/ | 176,246 |
| 47 | Single-Blind Method/ | 32,955 |
| 48 | clinical trial/ | 538,886 |
| 49 | randomized controlled trial/ | 600,970 |
| 50 | pragmatic clinical trial.pt. | 2250 |
| 51 | randomized controlled trial.pt. | 600,970 |
| 52 | Random Allocation/ | 106,969 |
| 53 | placebos/ | 35,932 |
| 54 | ((singl$ or doubl$ or treb$ or tripl$) adj (blind$3 or dumm$3 or mask$3)).ti,ab,kw. | 199,914 |
| 55 | trial.ti. | 294,062 |
| 56 | placebo$.ti,ab,kw. | 250,723 |
| 57 | (randomi?ed controlled trial$ or rct).ti,ab,kw. | 271,137 |
| 58 | randomi?ation.ti,ab,kw. | 52,344 |
| 59 | (crossover or cross over).ti,ab,kw. | 99,596 |
| 60 | (random$ adj2 allocat$).ti,ab,kw. | 44,515 |
| 61 | or/45-60 | 1,498,979 |
| 62 | 21 and 44 and 61 | 571 |
| 63 | animals/ not humans/ | 5,125,725 |
| 64 | 62 not 63 | 571 |
| 65 | Letter/ | 1,230,756 |
| 66 | editorial/ | 665,899 |
| 67 | Historical Article/ | 369,444 |
| 68 | Comment/ | 1,021,285 |
| 69 | Case Reports/ | 2,359,542 |
| 70 | (letter or comment$).ti. | 192,623 |
| 71 | (case reports or historical article or letter or comment or editorial).pt. | 4,670,213 |
| 72 | or/65-71 | 4,741,784 |
| 73 | 64 not 72 | 557 |
| 74 | limit 73 english language | 534 |
| 75 | limit 74 to ”r="2022 -Curr”nt" | 60 |

**eTable 1C. Search strategy for nmHSPC clinical review. Cochrane Central Register of Controlled Trials January 2022; EBM Reviews—Cochrane Database of Systematic Reviews 2005 to April 6, 2022. Search conducted on April 13, 2022.**

| **ID** | **Search string** | **# Hits** |
| --- | --- | --- |
| 1 | exp Prostatic Neoplasms/ | 6117 |
| 2 | (prostat$ adj6 (neoplas$ or canc$ or carcinoma$ or adeno$ or tumour$ or tumor$ or malignan$ or sarcoma$ or lymphoma$ or leiomyosarcoma$)).mp. | 17,163 |
| 3 | 1 or 2 | 17,163 |
| 4 | exp Neoplasms, Hormone-Dependent/ | 195 |
| 5 | ((hormon$ or androgen or castration$) adj2 (dependen$ or naiv$ or sensitiv$)).mp. | 1769 |
| 6 | (HSPC or HSPCa or ADPC or ADPCA or CSPC).mp. | 103 |
| 7 | Or/4-6 | 1831 |
| 8 | 3 and 7 | 902 |
| 9 | (earl$ or localised or localized or locally advance$ or non metasta$ or nonmetasta$ or not-metasta$ or M0 or stage$ one or stage$ 1 or stage$ I or stage$ two or stage$ 2 or stage$ II or stage$ 2A or stage$ IIA or stage$ 2B or stage$ IIB stage$ 2C or stage$ IIC or stage$ three or stage$ 3 or stage$ III or stage$ 3A or stage$ IIIA or stage$ 3B or IIIB stage$ 3C or stage$ IIIC or stage$ four or stage$ 4 or stage$ IV or stage$ 4A or stage$ IVA).mp. | 207,535 |
| 10 | 8 and 9 | 284 |
| 11 | (high$ risk$ adj10 (earl$ or localised or localized or locally advance$ or non metasta$ or nonmetasta$ or not-metasta$ or M0 or stage$ one or stage$ 1 or stage$ I or stage$ two or stage$ 2 or stage$ II or stage$ 2A or stage$ IIA or stage$ 2B or stage$ IIB stage$ 2C or stage$ IIC or stage$ three or stage$ 3 or stage$ III or stage$ 3A or stage$ IIIA or stage$ 3B or IIIB stage$ 3C or stage$ IIIC or stage$ four or stage$ 4 or stage$ IV or stage$ 4A or stage$ IVA)).mp. | 4165 |
| 12 | 3 and 11 | 482 |
| 13 | 10 or 12 | 715 |
| 14 | Prostate-Specific Antigen/ | 1395 |
| 15 | (rise$ or rising or increas$ or doubl$).mp. | 743,982 |
| 16 | 14 and 15 | 709 |
| 17 | ((prostat$ specific antigen$ or PSA) adj3 (rise$ or rising or increas$ or doubl$)).mp. | 1109 |
| 18 | (biochemical adj (failure or relapse)).mp. | 573 |
| 19 | Or/16-18 | 2071 |
| 20 | 3 and 9 and 19 | 799 |
| 21 | 13 or 20 | 1362 |
| 22 | watchful waiting/ | 304 |
| 23 | (Watch$ adj2 wait$).ti,ab,kw. | 868 |
| 24 | active surveillance.ti,ab,kw. | 796 |
| 25 | Conservative Treatment/ | 163 |
| 26 | ((careful$ or prostat$) adj2 monitor$).ti,ab,kw. | 952 |
| 27 | ((defer$ or delay$ or expectant$) adj2 (treatment$ or management$)).ti,ab,kw. | 3964 |
| 28 | ((observ$ or surveillance) adj3 prostat$).ti,ab,kw. | 322 |
| 29 | or/22-28 | 6968 |
| 30 | exp Prostatectomy/ | 1865 |
| 31 | (surger$ or adenectomy or resect$ or prostatectom$ or remov$).ti,ab,kw. | 250,401 |
| 32 | exp Lymph Node Excision/ | 1438 |
| 33 | lymph node dissection.ti,ab,kw. | 3107 |
| 34 | (PLND or RRP or RPP).ti,ab,kw. | 650 |
| 35 | or/30-34 | 251,724 |
| 36 | exp Radiotherapy/ | 6613 |
| 37 | (external beam adj3 (radiation$ or irradiation$ or RT or therap$)).ti,ab,kw. | 1061 |
| 38 | EBRT.ti,ab,kw. | 716 |
| 39 | ((stereotactic or stereotaxic) adj3 (radiation$ or radiotherap$)).ti,ab,kw. | 1406 |
| 40 | (SABR or SABRT or SBRT).ti,ab,kw. | 1055 |
| 41 | exp Proton Therapy/ | 56 |
| 42 | (proton beam adj3 (radiation$ or therap$)).ti,ab,kw. | 129 |
| 43 | PBRT.ti,ab,kw. | 14 |
| 44 | exp Radiotherapy, Conformal/ | 688 |
| 45 | (three dimensional conformal adj3 (radiation therap$ or radiotherap$)).ti,ab,kw. | 303 |
| 46 | 3d-CRT.ti,ab,kw. | 273 |
| 47 | exp Radiotherapy, Intensity-Modulated/ | 349 |
| 48 | (intensity modulated adj3 (therap$ or radiotherap$)).ti,ab,kw. | 1930 |
| 49 | (IGRT or IMRT).ti,ab,kw. | 1848 |
| 50 | exp Radiotherapy, Image-Guided/ | 91 |
| 51 | (image guided adj3 (radiation$ therap$ or radiotherap$)).ti,ab,kw. | 341 |
| 52 | exp Brachytherapy/ | 718 |
| 53 | brachytherap$.ti,ab,kw. | 2250 |
| 54 | or/36-53 | 12,482 |
| 55 | ((surger$ or surgical) adj3 castrat$).ti,ab,kw. | 251 |
| 56 | exp Orchiectomy/ | 342 |
| 57 | (orchectom$ or orcheotom$ or orchidectom$ or testectom$).ti,ab,kw. | 144 |
| 58 | or/55-57 | 633 |
| 59 | exp Antineoplastic Agents, Hormonal/ | 18,379 |
| 60 | (androgen deprivation therap$ or ADT$).mp. | 2581 |
| 61 | exp Gonadotropin-Releasing Hormone/ | 2702 |
| 62 | (lutein$ hormon$ releas$ or LHRH$ or LH RH$).mp. | 1518 |
| 63 | goserelin/ | 582 |
| 64 | (goserelin or ici 118 630 or ici 118630 or ici118630 or novimp or prozoladex or reseligo or zoladex or 65807-02-5).mp. | 1248 |
| 65 | (histrelin or orf 17070 or orf17070 or "rl 0903" or rl0903 or rwj 17070 or rwj17070 or spd 424 or spd424 or supprelin or vantas or "vp 002" or vp002 or 220810-26-4).mp. | 17 |
| 66 | leuprolide/ | 708 |
| 67 | (leuprorelin$ or a 43818 or a43818 or abbott 43818 or camcevi or carcinil or ckd 841 or ckd841 or daronda or depo lupron or eligard or eliprogel or elityran or enanton or enantone or fensolvi or ginecrin or klebrocid or la 2575 or la2575 or leptoprol or lerin or leuplin or leupro-sandoz or leuprogel or leuprolid or leuprolide$ or leupron or leuprone or leuprostin or lorelin or lucrin or lupride or luprolex or lupron or lutrate or ovarest or politrate or procrin or prostap or prostaplant or reliser or sixantone or sot 375 or sot375 or tap 144 or tap144 or tol 2506 or tol2506 or trenantone or viadur or vp 4896 or vp4896 or 53714-56-0 or 74381-53-6).mp. | 1455 |
| 68 | Triptorelin Pamoate/ | 468 |
| 69 | (triptorelin$ or arvekap or ay 25650 or ay25650 or bim 21003 or bim21003 or bn 52014 or bn52014 or cl 118532 or cl118532 or debio 8200 or debio 8206 or debio8200 or debio8206 or decapeptyl or detryptorelin or diphereline or fertipeptil or gonapeptyl or isr 48 or isr48 or microrelin or moapar or ovugel or pamorelin or salvacyl or spherotide or trelstar or triptodur or wy 424222 or wy42422 or 57773-63-4).mp. | 1047 |
| 70 | (degarelix or fe 200486 or fe200486 or firmagon or 214766-78-6).mp. | 228 |
| 71 | (relugolix or mvt 601 or mvt601 or orgovyx or relumina or rvt 601 or rvt601 or t 1331285 or t1331285 or tak 385 or tak385 or 737789-87-6).mp. | 92 |
| 72 | exp Androgen Antagonists/ | 1496 |
| 73 | (androgen antagonist$ or anti androgen$ or antiandrogen$).mp. | 1900 |
| 74 | (bicalutamide or biluron or casodex or cosudex or ici 176334 or lutamidal or probic or raffolutil or 90357-06-5).mp. | 585 |
| 75 | Flutamide/ | 340 |
| 76 | (flutamide or apimid or cytamid or drogenil or etaconil or euflex or eulexin or eulexine or flucinom or fludinom or flugerel or fluken or flulem or flumid or flutamex or flutamin or flutan or flutaplex or flutax or flutol or fluxus or fugerel or niftolid or niftolide or niphtholid or odyne or prostamid or prostica or prostogenat or sch 13521 or sch13521 or sebatrol or tafenil or testac or 13311-84-7).mp. | 593 |
| 77 | (anadron or anandron or canandron or nilandron or nitulamide or rn 23908 or ru 23908 or 63612-50-0).mp. | 39 |
| 78 | (enzalutamide or mdv 3100 or mdv3100 or xtandi or 915087-33-1).mp. | 899 |
| 79 | (apalutamide or arn 509 or arn509 or erleada or 956104-40-8).mp. | 208 |
| 80 | (darolutamide or bay 1841788 or bay1841788 or nubeqa or odm 201 or odm201 or 1297538 32 9).mp. | 133 |
| 81 | or/59-80 | 25,759 |
| 82 | 29 or 35 or 54 or 58 or 81 | 287,280 |
| 83 | 21 and 82 | 1142 |
| 84 | limit 83 to English language [Limit not valid in CDSR; records were retained] | 1094 |
| 85 | limit 84 to yr="2012 -Current" | 851 |
| 86 | remove duplicates from 85 | 833 |

**eTable 1D. Search strategy for nmHSPC clinical reviews. EBM Reviews—Cochrane Central Register of Controlled Trials August 2023 and Cochrane Database of Systematic Reviews 2005 to September 27, 2023. Search conducted on October 3, 2023.**

| **ID** | **Search string** | **# Hits** |
| --- | --- | --- |
| 1 | exp Prostatic Neoplasms/ | 7562 |
| 2 | (prostat$ adj6 (neoplas$ or canc$ or carcinoma$ or adeno$ or tumour$ or tumor$ or malignan$ or sarcoma$ or lymphoma$ or leiomyosarcoma$)).mp. | 17,792 |
| 3 | 1 or 2 | 17,792 |
| 4 | exp Neoplasms, Hormone-Dependent/ | 261 |
| 5 | ((hormon$ or androgen or castration$) adj2 (dependen$ or naiv$ or sensitiv$)).mp. | 2014 |
| 6 | (HSPC or HSPCa or ADPC or ADPCA or CSPC).mp. | 119 |
| 7 | or/4-6 | 2080 |
| 8 | 3 and 7 | 1079 |
| 9 | (earl$ or localised or localized or locally advance$ or non metasta$ or nonmetasta$ or not-metasta$ or M0 or stage$ one or stage$ 1 or stage$ I or stage$ two or stage$ 2 or stage$ II or stage$ 2A or stage$ IIA or stage$ 2B or stage$ IIB stage$ 2C or stage$ IIC or stage$ three or stage$ 3 or stage$ III or stage$ 3A or stage$ IIIA or stage$ 3B or IIIB stage$ 3C or stage$ IIIC or stage$ four or stage$ 4 or stage$ IV or stage$ 4A or stage$ IVA).mp. | 220,111 |
| 10 | 8 and 9 | 327 |
| 11 | (high$ risk$ adj10 (earl$ or localised or localized or locally advance$ or non metasta$ or nonmetasta$ or not-metasta$ or M0 or stage$ one or stage$ 1 or stage$ I or stage$ two or stage$ 2 or stage$ II or stage$ 2A or stage$ IIA or stage$ 2B or stage$ IIB stage$ 2C or stage$ IIC or stage$ three or stage$ 3 or stage$ III or stage$ 3A or stage$ IIIA or stage$ 3B or IIIB stage$ 3C or stage$ IIIC or stage$ four or stage$ 4 or stage$ IV or stage$ 4A or stage$ IVA)).mp. | 4556 |
| 12 | 3 and 11 | 527 |
| 13 | 10 or 12 | 800 |
| 14 | Prostate-Specific Antigen/ | 1734 |
| 15 | (rise$ or rising or increas$ or doubl$).mp. | 782,650 |
| 16 | 14 and 15 | 834 |
| 17 | ((prostat$ specific antigen$ or PSA) adj3 (rise$ or rising or increas$ or doubl$)).mp. | 1171 |
| 18 | (biochemical adj (failure or relapse)).mp. | 600 |
| 19 | or/16-18 | 2239 |
| 20 | 3 and 9 and 19 | 872 |
| 21 | 13 or 20 | 1502 |
| 22 | exp Antineoplastic Agents, Hormonal/ | 20,387 |
| 23 | (androgen deprivation therap$ or ADT$).mp. | 2892 |
| 24 | exp Gonadotropin-Releasing Hormone/ | 2968 |
| 25 | (lutein$ hormon$ releas$ or LHRH$ or LH RH$).mp. | 1578 |
| 26 | goserelin/ | 628 |
| 27 | (goserelin or ici 118 630 or ici 118630 or ici118630 or novimp or prozoladex or reseligo or zoladex or 65807-02-5).mp. | 1289 |
| 28 | (histrelin or orf 17070 or orf17070 or "rl 0903" or rl0903 or rwj 17070 or rwj17070 or spd 424 or spd424 or supprelin or vantas or "vp 002" or vp002 or 220810-26-4).mp. | 19 |
| 29 | leuprolide/ | 749 |
| 30 | (leuprorelin$ or a 43818 or a43818 or abbott 43818 or camcevi or carcinil or ckd 841 or ckd841 or daronda or depo lupron or eligard or eliprogel or elityran or enanton or enantone or fensolvi or ginecrin or klebrocid or la 2575 or la2575 or leptoprol or lerin or leuplin or leupro-sandoz or leuprogel or leuprolid or leuprolide$ or leupron or leuprone or leuprostin or lorelin or lucrin or lupride or luprolex or lupron or lutrate or ovarest or politrate or procrin or prostap or prostaplant or reliser or sixantone or sot 375 or sot375 or tap 144 or tap144 or tol 2506 or tol2506 or trenantone or viadur or vp 4896 or vp4896 or 53714-56-0 or 74381-53-6).mp. | 1510 |
| 31 | Triptorelin Pamoate/ | 497 |
| 32 | (triptorelin$ or arvekap or ay 25650 or ay25650 or bim 21003 or bim21003 or bn 52014 or bn52014 or cl 118532 or cl118532 or debio 8200 or debio 8206 or debio8200 or debio8206 or decapeptyl or detryptorelin or diphereline or fertipeptil or gonapeptyl or isr 48 or isr48 or microrelin or moapar or ovugel or pamorelin or salvacyl or spherotide or trelstar or triptodur or wy 424222 or wy42422 or 57773-63-4).mp. | 1103 |
| 33 | (degarelix or fe 200486 or fe200486 or firmagon or 214766-78-6).mp. | 236 |
| 34 | (relugolix or mvt 601 or mvt601 or orgovyx or relumina or rvt 601 or rvt601 or t 1331285 or t1331285 or tak 385 or tak385 or 737789-87-6).mp. | 138 |
| 35 | exp Androgen Antagonists/ | 1798 |
| 36 | (androgen antagonist$ or anti androgen$ or antiandrogen$).mp. | 2159 |
| 37 | (bicalutamide or biluron or casodex or cosudex or ici 176334 or lutamidal or probic or raffolutil or 90357-06-5).mp. | 596 |
| 38 | Flutamide/ | 359 |
| 39 | (flutamide or apimid or cytamid or drogenil or etaconil or euflex or eulexin or eulexine or flucinom or fludinom or flugerel or fluken or flulem or flumid or flutamex or flutamin or flutan or flutaplex or flutax or flutol or fluxus or fugerel or niftolid or niftolide or niphtholid or odyne or prostamid or prostica or prostogenat or sch 13521 or sch13521 or sebatrol or tafenil or testac or 13311-84-7).mp. | 595 |
| 40 | (anadron or anandron or canandron or nilandron or nitulamide or rn 23908 or ru 23908 or 63612-50-0).mp. | 39 |
| 41 | (enzalutamide or mdv 3100 or mdv3100 or xtandi or 915087-33-1).mp. | 991 |
| 42 | (apalutamide or arn 509 or arn509 or erleada or 956104-40-8).mp. | 269 |
| 43 | (darolutamide or bay 1841788 or bay1841788 or nubeqa or odm 201 or odm201 or 1297538 32 9).mp. | 209 |
| 44 | or/22-43 | 28,342 |
| 45 | 21 and 44 | 918 |
| 46 | limit 45 to english language [Limit not valid in CDSR; records were retained] | 909 |
| 47 | limit 46 to yr="2022 -Current" | 117 |
| 48 | remove duplicates from 47 | 116 |

**eTable 1E. Search strategy for nmHSPC clinical reviews. Embase 1974 to October 2, 2023. Search conducted on October 3, 2023.**

| **ID** | **Search string** | **# Hits** |
| --- | --- | --- |
| 1 | exp prostate tumor/ | 296,347 |
| 2 | (prostat$ adj6 (neoplas$ or canc$ or carcinoma$ or adeno$ or tumour$ or tumor$ or malignan$ or sarcoma$ or lymphoma$ or leiomyosarcoma$)).mp. | 339,941 |
| 3 | 1 or 2 | 339,942 |
| 4 | exp hormone-dependent neoplasm/ | 63 |
| 5 | ((hormon$ or androgen or castration$) adj2 (dependen$ or naiv$ or sensitiv$)).mp. | 34,519 |
| 6 | (HSPC or HSPCa or ADPC or ADPCA or CSPC).mp. | 5197 |
| 7 | or/4-6 | 39,290 |
| 8 | 3 and 7 | 11,947 |
| 9 | (earl$ or localised or localized or locally advance$ or non metasta$ or nonmetasta$ or not-metasta$ or M0 or stage$ one or stage$ 1 or stage$ I or stage$ two or stage$ 2 or stage$ II or stage$ 2A or stage$ IIA or stage$ 2B or stage$ IIB stage$ 2C or stage$ IIC or stage$ three or stage$ 3 or stage$ III or stage$ 3A or stage$ IIIA or stage$ 3B or IIIB stage$ 3C or stage$ IIIC or stage$ four or stage$ 4 or stage$ IV or stage$ 4A or stage$ IVA).mp. | 3,631,787 |
| 10 | 8 and 9 | 2440 |
| 11 | (high$ risk$ adj10 (earl$ or localised or localized or locally advance$ or non metasta$ or nonmetasta$ or not-metasta$ or M0 or stage$ one or stage$ 1 or stage$ I or stage$ two or stage$ 2 or stage$ II or stage$ 2A or stage$ IIA or stage$ 2B or stage$ IIB stage$ 2C or stage$ IIC or stage$ three or stage$ 3 or stage$ III or stage$ 3A or stage$ IIIA or stage$ 3B or IIIB stage$ 3C or stage$ IIIC or stage$ four or stage$ 4 or stage$ IV or stage$ 4A or stage$ IVA)).mp. | 41,714 |
| 12 | 3 and 11 | 2966 |
| 13 | 10 or 12 | 5220 |
| 14 | exp prostate specific antigen/ | 70,335 |
| 15 | (rise$ or rising or increas$ or doubl$).mp. | 9,932,400 |
| 16 | 14 and 15 | 21,882 |
| 17 | ((prostat$ specific antigen$ or PSA) adj3 (rise$ or rising or increas$ or doubl$)).mp. | 10,388 |
| 18 | (biochemical adj (failure or relapse)).mp. | 6639 |
| 19 | or/16-18 | 30,675 |
| 20 | 3 and 9 and 19 | 9760 |
| 21 | 13 or 20 | 13,879 |
| 22 | (histrelin or orf 17070 or orf17070 or "rl 0903" or rl0903 or rwj 17070 or rwj17070 or spd 424 or spd424 or supprelin or vantas or "vp 002" or vp002 or 220810-26-4).mp. | 579 |
| 23 | exp leuprorelin/ | 13,272 |
| 24 | (leuprorelin$ or a 43818 or a43818 or abbott 43818 or camcevi or carcinil or ckd 841 or ckd841 or daronda or depo lupron or eligard or eliprogel or elityran or enanton or enantone or fensolvi or ginecrin or klebrocid or la 2575 or la2575 or leptoprol or lerin or leuplin or leupro-sandoz or leuprogel or leuprolid or leuprolide$ or leupron or leuprone or leuprostin or lorelin or lucrin or lupride or luprolex or lupron or lutrate or ovarest or politrate or procrin or prostap or prostaplant or reliser or sixantone or sot 375 or sot375 or tap 144 or tap144 or tol 2506 or tol2506 or trenantone or viadur or vp 4896 or vp4896 or 53714-56-0 or 74381-53-6).mp. | 13,651 |
| 25 | exp triptorelin/ | 6629 |
| 26 | (triptorelin$ or arvekap or ay 25650 or ay25650 or bim 21003 or bim21003 or bn 52014 or bn52014 or cl 118532 or cl118532 or debio 8200 or debio 8206 or debio8200 or debio8206 or decapeptyl or detryptorelin or diphereline or fertipeptil or gonapeptyl or isr 48 or isr48 or microrelin or moapar or ovugel or pamorelin or salvacyl or spherotide or trelstar or triptodur or wy 424222 or wy42422 or 57773-63-4).mp. | 6830 |
| 27 | 13 or 20 | 13,879 |
| 28 | exp "antineoplastic hormone agonists and antagonists"/ | 657,686 |
| 29 | (androgen deprivation therap$ or ADT$).mp. | 26,233 |
| 30 | exp gonadorelin/ | 41,024 |
| 31 | (lutein$ hormon$ releas$ or LHRH$ or LH RH$).mp. | 22,649 |
| 32 | exp goserelin/ | 8086 |
| 33 | (degarelix or fe 200486 or fe200486 or firmagon or 214766-78-6).mp. | 1273 |
| 34 | (relugolix or mvt 601 or mvt601 or orgovyx or relumina or rvt 601 or rvt601 or t 1331285 or t1331285 or tak 385 or tak385 or 737789-87-6).mp. | 364 |
| 35 | exp antiandrogen/ | 78,580 |
| 36 | (androgen antagonist$ or anti androgen$ or antiandrogen$).mp. | 21,533 |
| 37 | (bicalutamide or biluron or casodex or cosudex or ici 176334 or lutamidal or probic or raffolutil or 90357-06-5).mp. | 7957 |
| 38 | exp flutamide/ | 8927 |
| 39 | (flutamide or apimid or cytamid or drogenil or etaconil or euflex or eulexin or eulexine or flucinom or fludinom or flugerel or fluken or flulem or flumid or flutamex or flutamin or flutan or flutaplex or flutax or flutol or fluxus or fugerel or niftolid or niftolide or niphtholid or odyne or prostamid or prostica or prostogenat or sch 13521 or sch13521 or sebatrol or tafenil or testac or 13311-84-7).mp. | 9359 |
| 40 | (anadron or anandron or canandron or nilandron or nitulamide or rn 23908 or ru 23908 or 63612-50-0).mp. | 355 |
| 41 | (enzalutamide or mdv 3100 or mdv3100 or xtandi or 915087-33-1).mp. | 10,637 |
| 42 | (apalutamide or arn 509 or arn509 or erleada or 956104-40-8).mp. | 1804 |
| 43 | (darolutamide or bay 1841788 or bay1841788 or nubeqa or odm 201 or odm201 or 1297538 32 9).mp. | 1016 |
| 44 | or/22-43 | 767,243 |
| 45 | exp randomized controlled trial/ | 786,840 |
| 46 | exp Double blind procedure/ | 211,035 |
| 47 | exp Single blind procedure/ | 51,896 |
| 48 | clinical trial/ | 1,070,719 |
| 49 | exp Randomization/ | 98,829 |
| 50 | (clinical adj1 trial$).ti,ab,kw. | 700,326 |
| 51 | exp placebo/ | 402,821 |
| 52 | ((singl$ or doubl$ or treb$ or tripl$) adj (blind$3 or dumm$3 or mask$3)).ti,ab,kw. | 282,364 |
| 53 | trial.ti. | 402,529 |
| 54 | placebo$.ti,ab,kw. | 368,686 |
| 55 | (randomi?ed controlled trial$ or rct).ti,ab,kw. | 361,933 |
| 56 | randomi?ation.ti,ab,kw. | 81,795 |
| 57 | (crossover or cross over).ti,ab,kw. | 124,921 |
| 58 | (random$ adj2 allocat$).ti,ab,kw. | 55,120 |
| 59 | or/45-58 | 2,566,978 |
| 60 | 21 and 44 and 59 | 3336 |
| 61 | animals/ not humans/ | 1,079,351 |
| 62 | 60 not 61 | 3335 |
| 63 | Letter/ | 1,211,777 |
| 64 | editorial/ | 749,582 |
| 65 | "history of medicine"/ | 41,049 |
| 66 | Case Report/ | 2,919,369 |
| 67 | (letter or comment$).ti. | 237,421 |
| 68 | (case reports or historical article or letter or comment or editorial).pt. | 2,071,718 |
| 69 | or/63-68 | 4,961,692 |
| 70 | 62 not 69 | 3272 |
| 71 | limit 70 to english language | 3192 |
| 72 | limit 71 to yr="2012 -Current" | 2152 |
| 73 | conference.so. | 670,739 |
| 74 | conference abstract.pt. | 4,899,897 |
| 75 | or/73-74 | 4,913,669 |
| 76 | limit 75 to yr="2012 -2020" | 3,317,501 |
| 77 | 72 not 76 | 1241 |

**eTable 2. Study designs across included studies**

| Study name (trial name) | Phase | Randomization (yes/no) | Blinding status | Crossover allowed (yes/no) | Single/multicenter | Geography |
| --- | --- | --- | --- | --- | --- | --- |
| EMBARK (14, 15, 25, 26) | 3 | Yes | Quadruple | No | Multicenter | Multinational |
| Autio, 2021 (NA) (27) | 2 | Yes | Open label | No | Multicenter | USA |
| Crook, 2012 (SWOG-JPR7) (23) | 3 | Yes | Open label | No | Multicenter | Canada |
| Duchesne, 2016  (TOAD) (24) | 3 | Yes | Open label | N/A | Multicenter | Australia, New Zealand, Canada |
| Morris, 2021 (TAX3503) (30) | 3 | Yes | Open label | No | Multicenter | USA, Belgium, Canada, Czech Republic, Germany, Lithuania, Poland, Slovakia, Spain |
| Oudard, 2019 (AOM 03108) (42) | 3 | Yes | Open label | N/A | Multicenter | France |
| Spetsieris 2021 (FINITE) (31, 43) | 2 | Yes | Open label | Yes | Single center | USA |
| Josefsson 2023 (44) | 3 | Yes | Open label | N/A | Multicenter | Sweden, Denmark, Finland, Netherlands |
| Aggarwal 2023 (PRESTO, AFT-19) (32) | 3 | Yes | Open label | N/A | Single center | USA |
| NCT01790126 (45) | 2 | Yes | Open label | N/A | Single center | USA |

**Abbreviations:** N/A: not applicable; USA: United States of America.

**eTable 3. Outcome availability across included studies**

| Author, year (trial name) | MFS | OS | Time to PSA progression | Time to castration resistance | % of patients with undetectable PSA | Grade ≥3* TRAEs (%) |
| --- | --- | --- | --- | --- | --- | --- |
| EMBARK^14, 15, 25, 26^ | **✓** (KM curve, N at risk, median MFS, HR, CI) | **✓** (KM curve, N at risk, median OS, HR, CI) | **✓** (KM curve, N at risk, median time to PSA progression, HR, CI) | **✓** (KM curve, N at risk, median time to castration resistance, HR, CI) | **✓** (n, %) | **✓** (n, %) |
| Autio, 2021 (NA)^27^ | **🗶** | **🗶** | **✓** (KM curve, N at risk, median time to PSA progression^¥^) | **🗶** | **✓**  (n, %) | **🗶** |
| Crook, 2012 (SWOG-JPR7)^23^ | **🗶** | **✓** (KM curve, N at risk, median OS, HR, CI) | **🗶** | **✓** (KM curve, N at risk, HR, CI) | **🗶** | **🗶** |
| Duchesne, 2016 (TOAD)^24^ | **🗶** | **✓** (KM curve, N at risk, HR, CI) | **🗶** | **✓** (HR, CI) | **🗶** | **🗶**^a^ |
| Morris, 2021 (TAX3503)^30^ | **🗶** | **✓** (KM curve, N at risk, HR, CI) | **✓** (KM curve, N at risk, HR, CI) | **🗶** | **🗶** | **✓** (n, %) |
| Oudard, 2019 (AOM 03108)^43^ | **✓** (KM curve, N at risk, median MFS, HR, CI) | **✓** (KM curve, N at risk, HR, CI) | **✓**  (KM curve, N at risk, median time to PSA progression, HR, CI) | **🗶** | **🗶** | **✓** (n, %) |
| Spetsieris, 2021 (FINITE)^31, 44^ | **🗶** | **🗶** | **✓** (KM curve, median time to PSA progression, HR, CI) | **🗶** | **🗶** | **🗶** |
| Josefsson 2023 (SPCG-14)^45^ | **🗶** | **🗶** | **✓** (KM curve, N at risk, median time to PSA progression) | **🗶** | **🗶** | **🗶** |
| Aggarwal 2023 (PRESTO, AFT-19)^32^ | **🗶** | **🗶** | **✓** (median time to PSA progression, HR, CI) | **🗶** | **🗶** | **🗶** |
| NCT01790126^46^ | **🗶** | **🗶** | **✓** (median time to PSA progression) | **🗶** | **🗶** | **🗶** |

**Abbreviations:** CI: confidence interval; HR: hazard ratio; KM: Kaplan–Meier; OS: overall survival; MFS: metastasis-free survival; PSA: prostate-specific antigen; TRAE: treatment-related adverse event.

^a^Results were available for the overall population only (instead of being reported per treatment arm).

**eTable 4. Risk-of-bias assessment across primary outcomes in included studies**

| **Author, year (trial name)** | **Outcome** | **D1** | **D2** | **D3** | **D4** | **D5** | **Overall** |  |  |  |
| --- | --- | --- | --- | --- | --- | --- | --- | --- | --- | --- |
| **Efficacy outcomes** | | | | | | | |  | **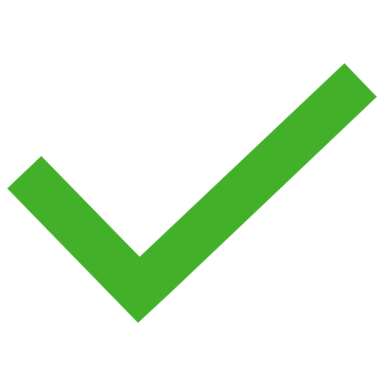** | Low risk |
| EMBARK | MFS | 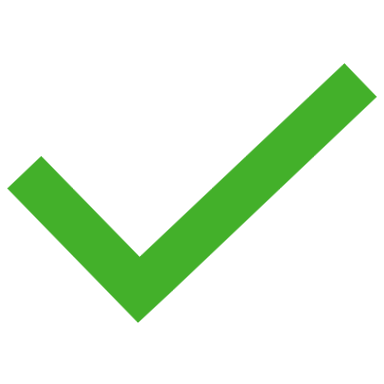 | 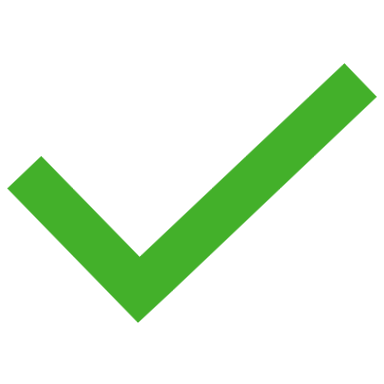 | 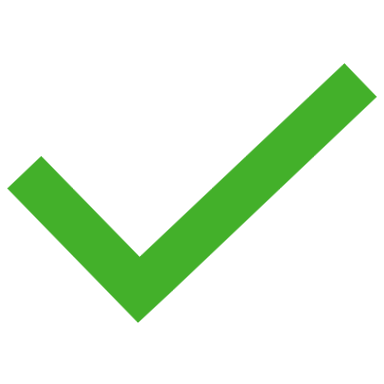 | 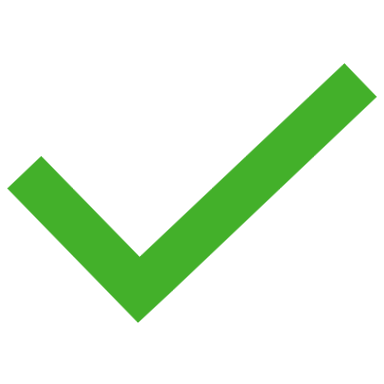 | 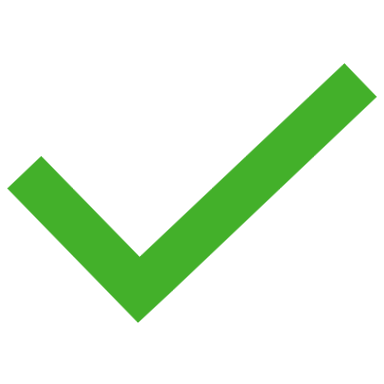 | 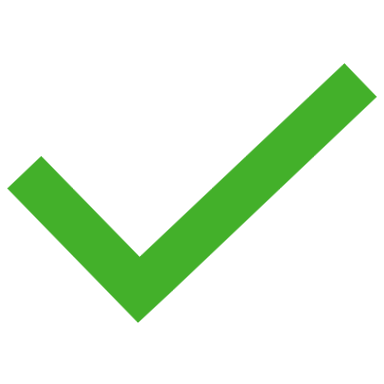 |  | **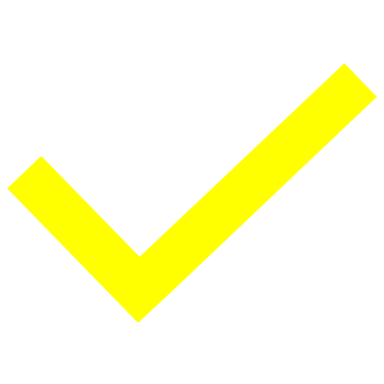** | Some concerns |
| Autio, 2021 (NR) | Time to PSA progression | **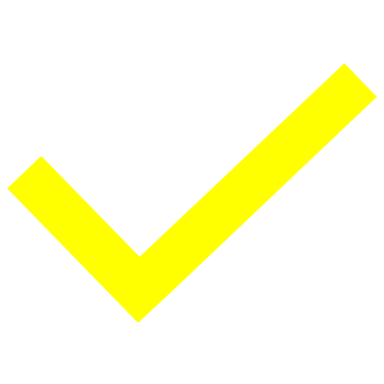** | **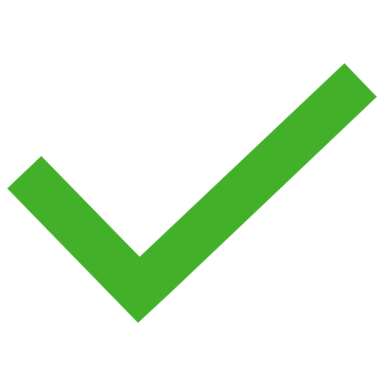** | **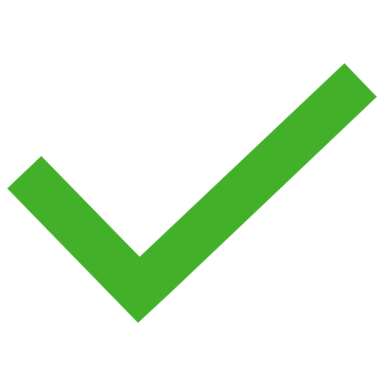** | **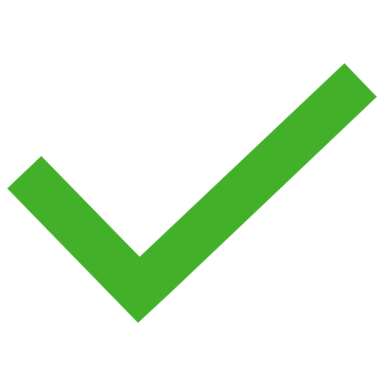** | **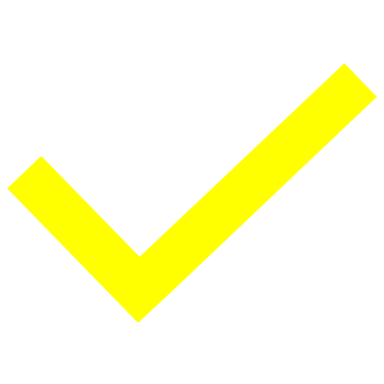** | **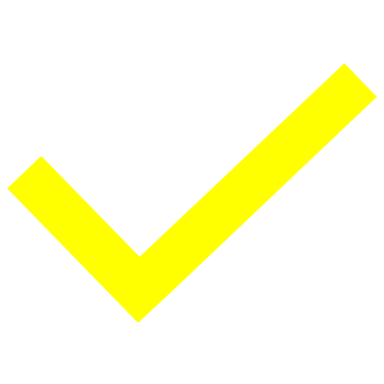** |  | **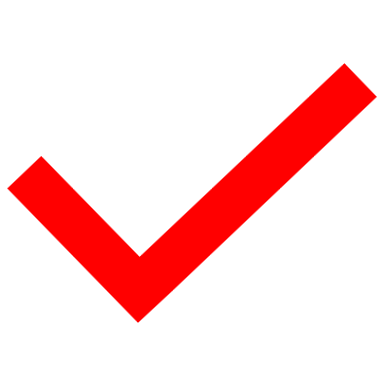** | High risk |
| Crook, 2012 (SWOG-JPR7) | OS | **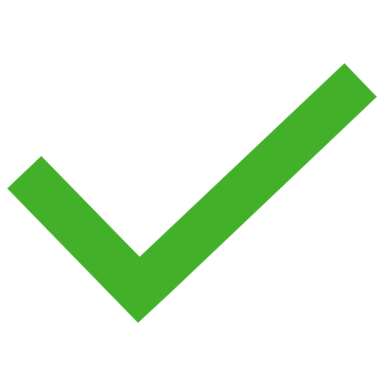** | **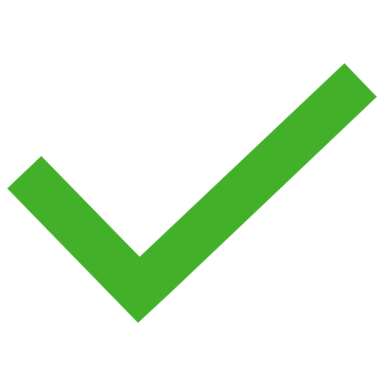** | **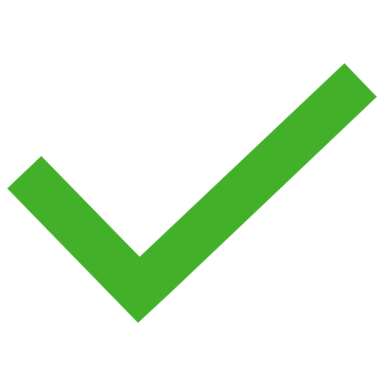** | **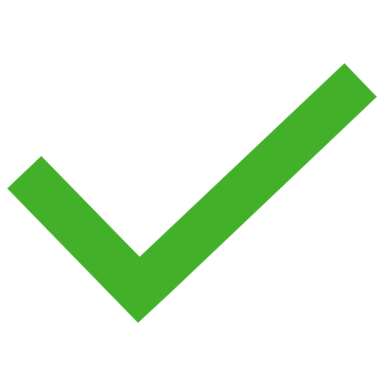** | **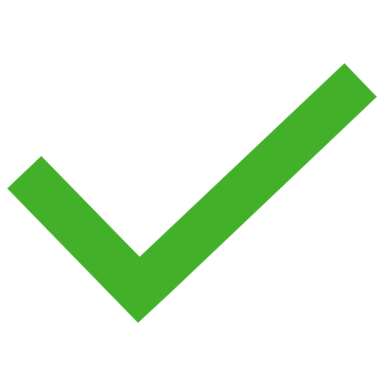** | **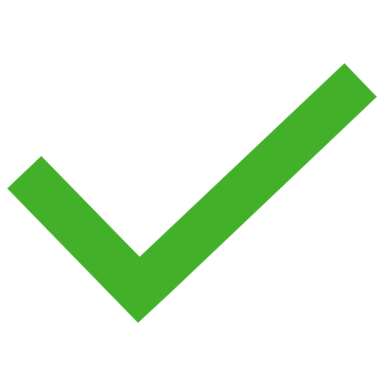** |  | D1 | Randomization process |
| Crook, 2012 (SWOG-JPR7) | Time to castration resistance | **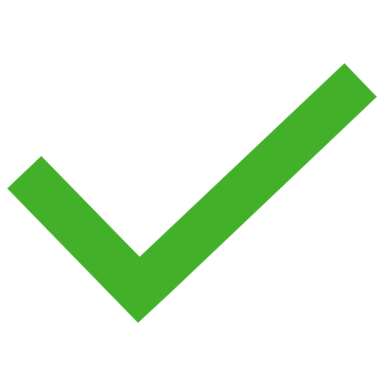** | **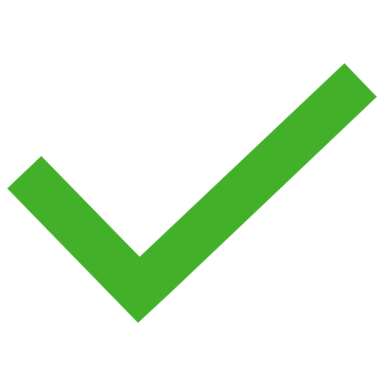** | **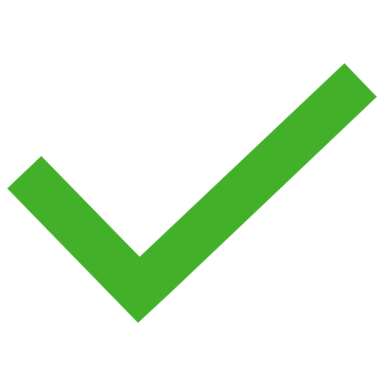** | **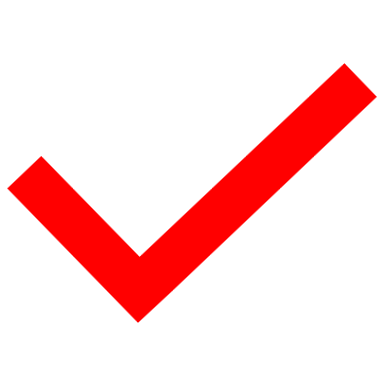** | **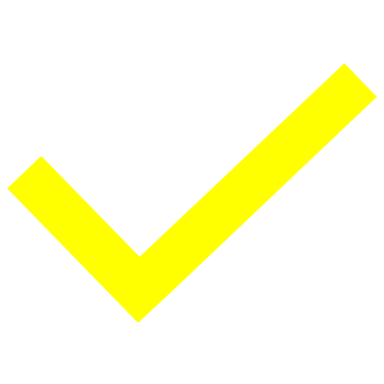** | **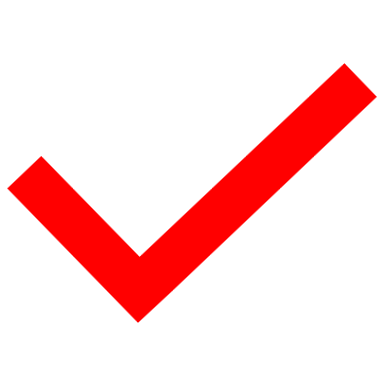** |  | D2 | Deviations from the intended interventions |
| Morris, 2021 (TAX3503) | Time to PSA progression | **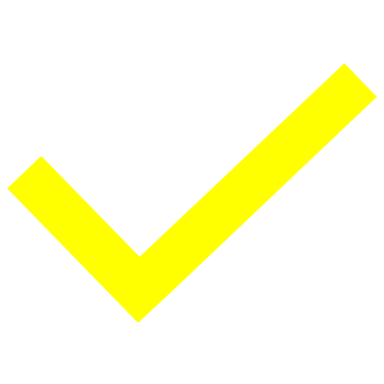** | **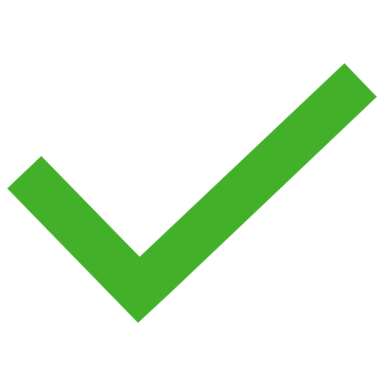** | **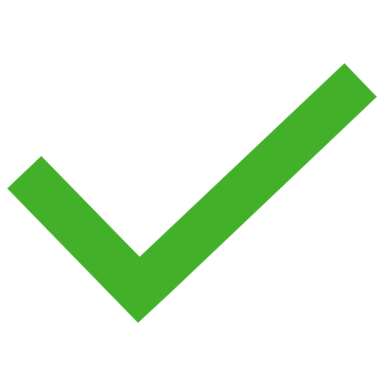** | **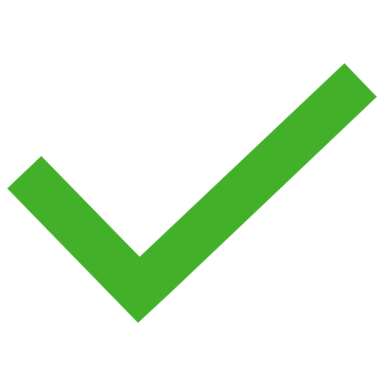** | **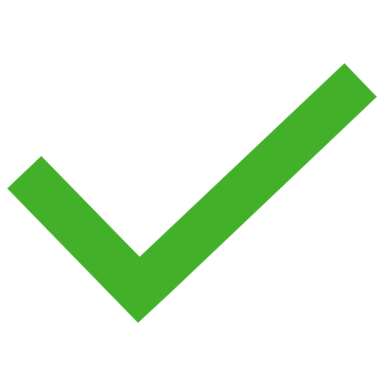** | **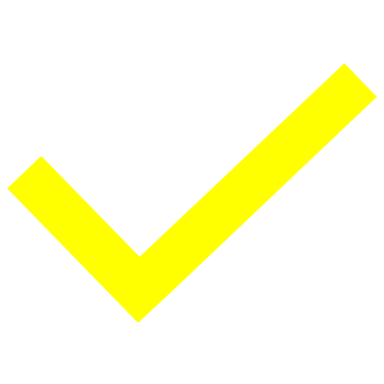** |  | D3 | Missing outcome data |
| Morris, 2021 (TAX3503) | OS | **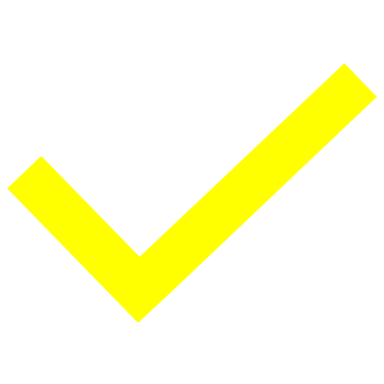** | **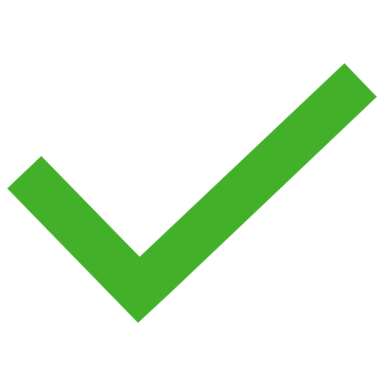** | **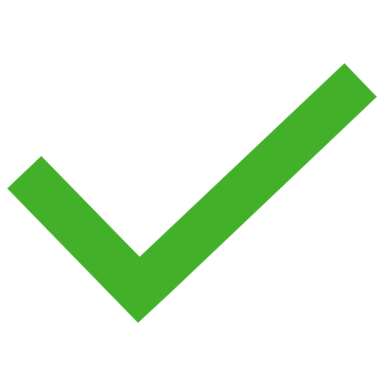** | **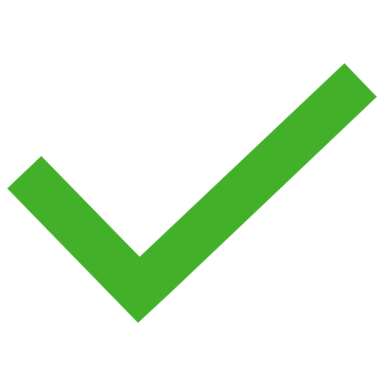** | **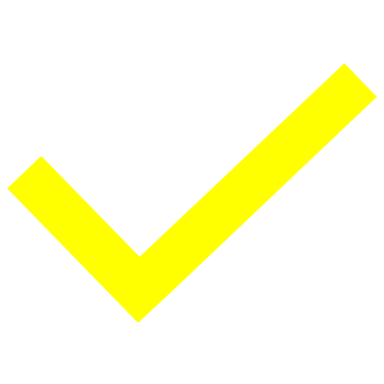** | **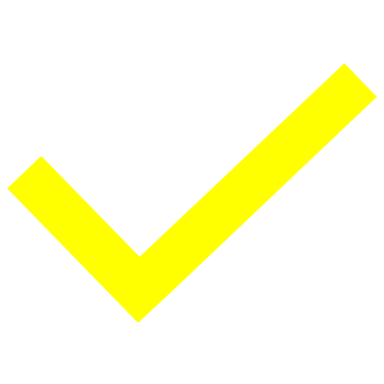** |  | D4 | Measurement of the outcome |
| Oudard, 2019 (AOM 03108) | OS | **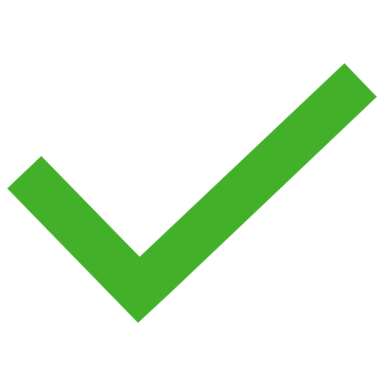** | **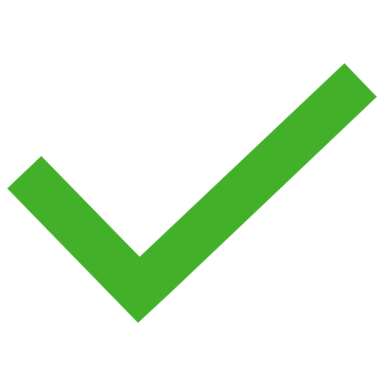** | **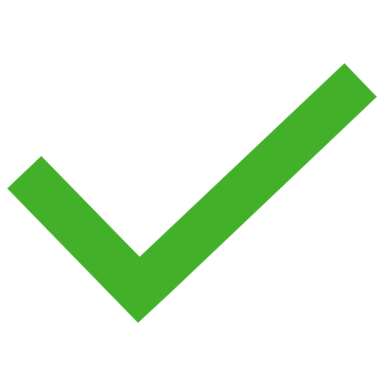** | **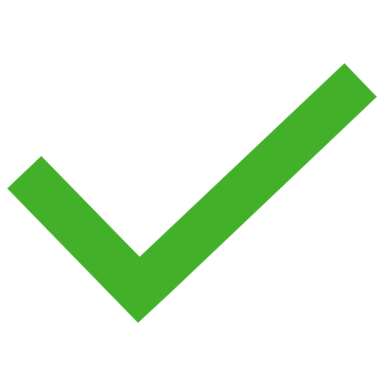** | **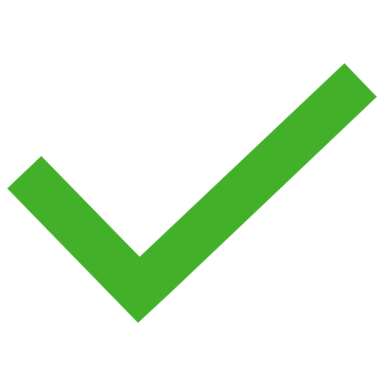** | **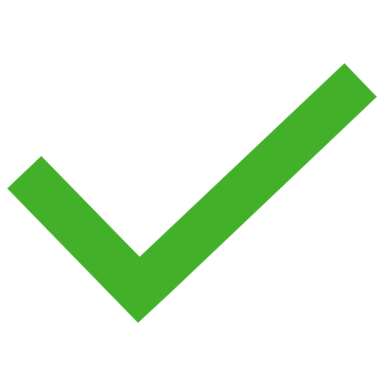** |  | D5 | Selection of the reported result |
| Oudard, 2019 (AOM 03108) | Time to PSA progression | **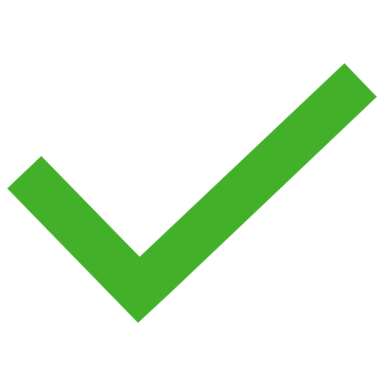** | **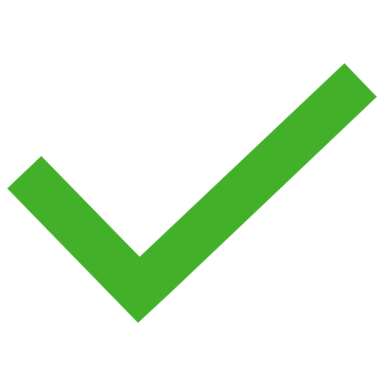** | **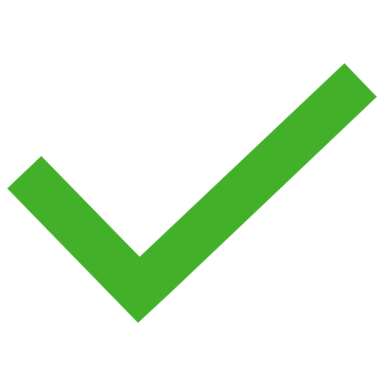** | **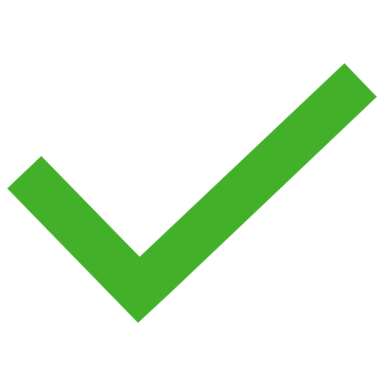** | **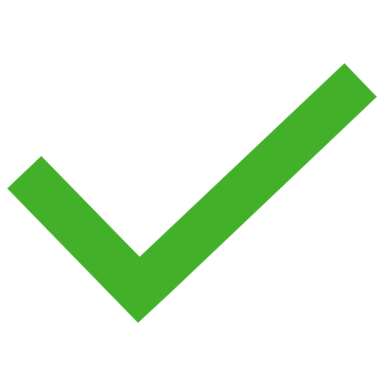** | **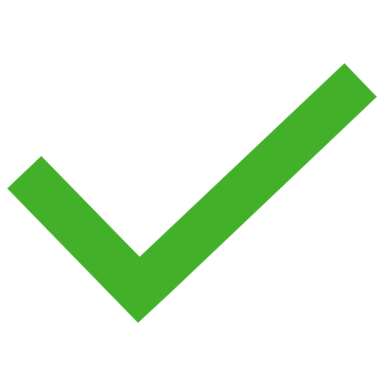** |  |  |  |
| Oudard, 2019 (AOM 03108) | MFS | **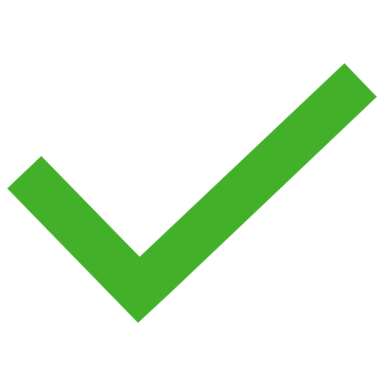** | **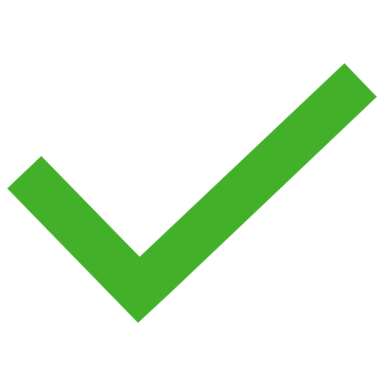** | **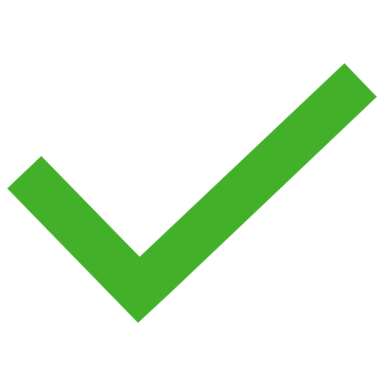** | **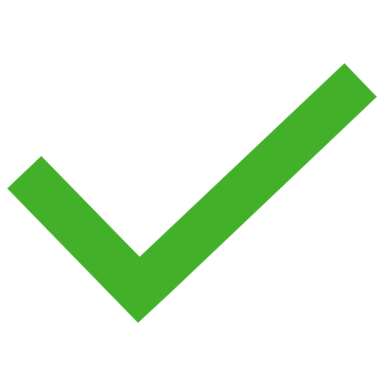** | **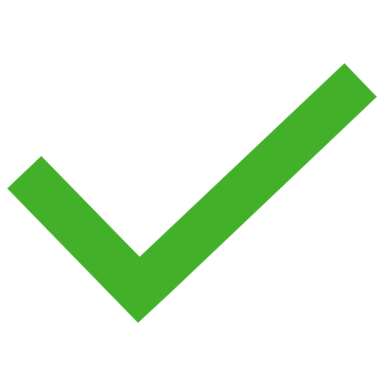** | **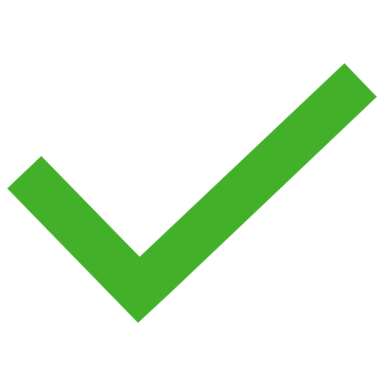** |  |  |  |
| Duchesne 2016 (TOAD)^25^ | OS | **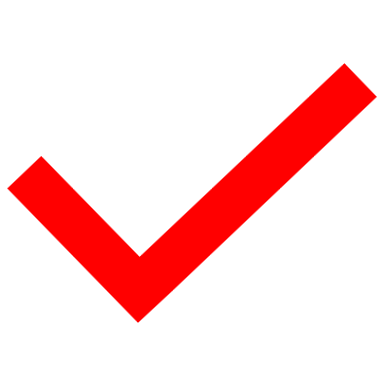** | **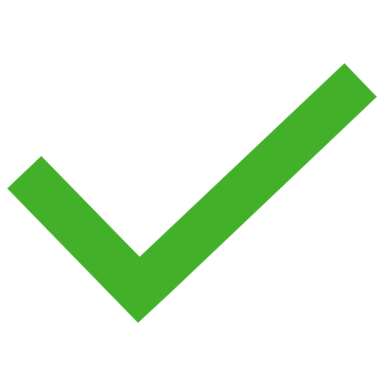** | **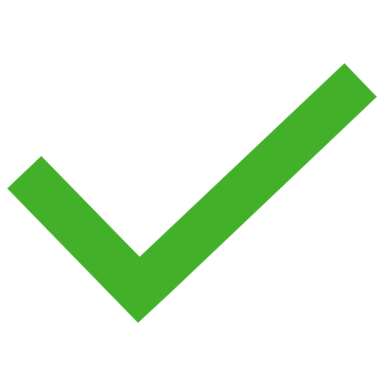** | **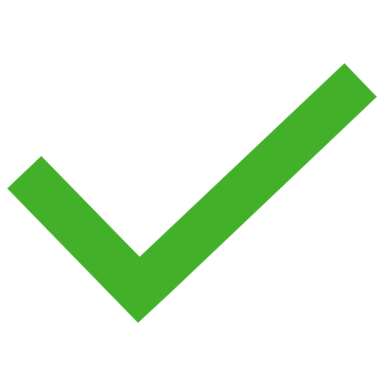** | **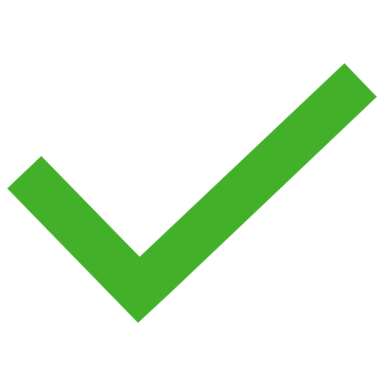** | **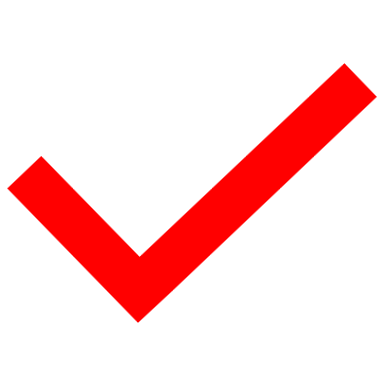** |  |  |  |
| Spetsieris, 2021 (FINITE) | Time to PSA progression | **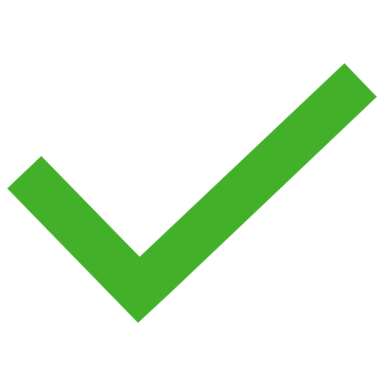** | **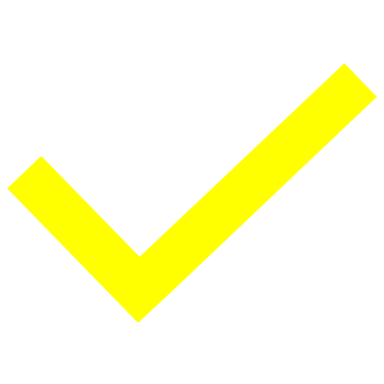** | **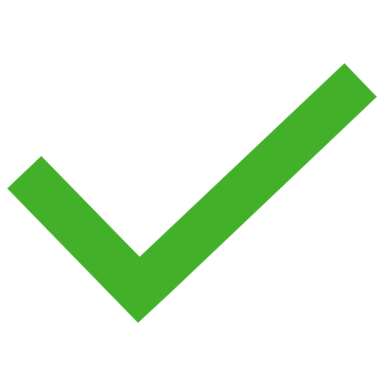** | **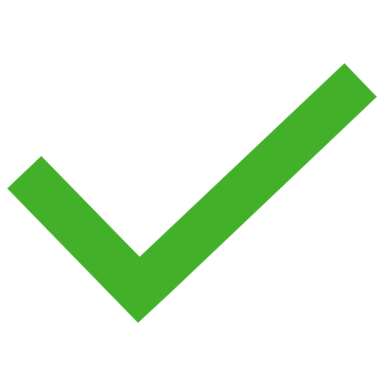** | **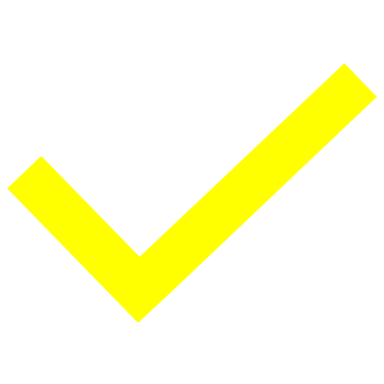** | **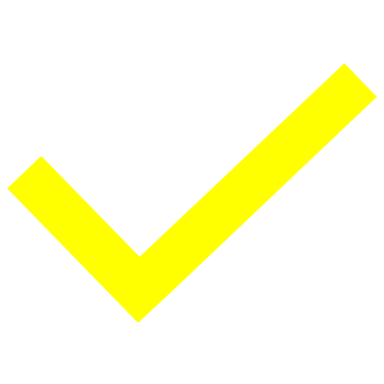** |  |  |  |
| Josefsson 2023 (SPCG-14) | Time to PSA progression | **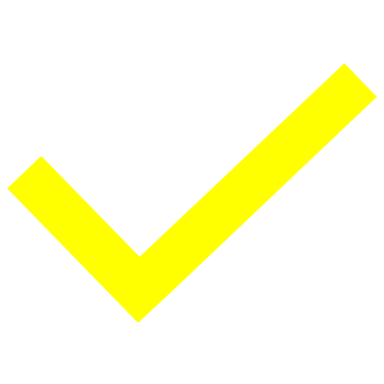** | **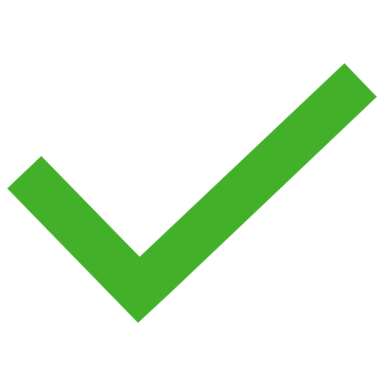** | **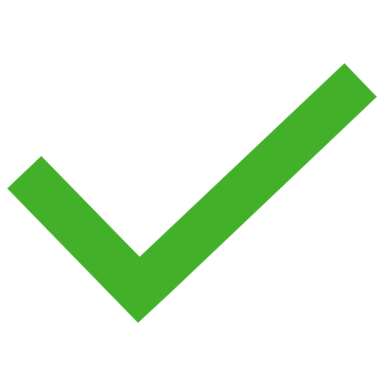** | **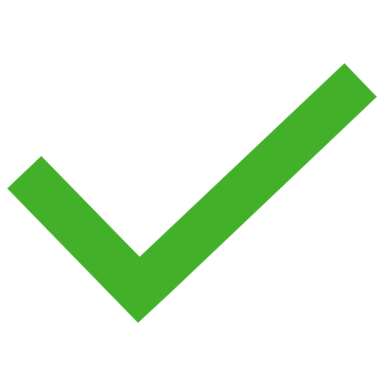** | **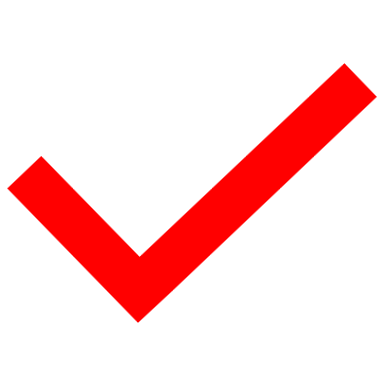** | **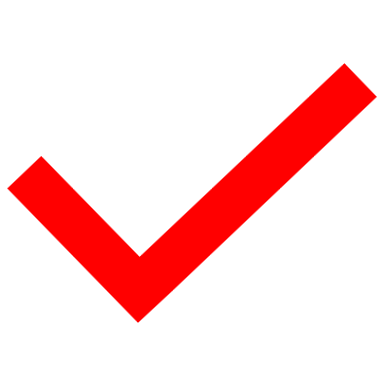** |  |  |  |
| Aggarwal 2023 (PRESTO AFT-19) | Time to PSA progression | **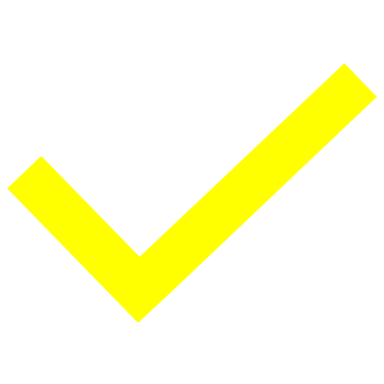** | **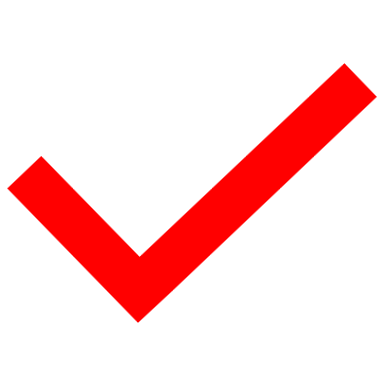** | **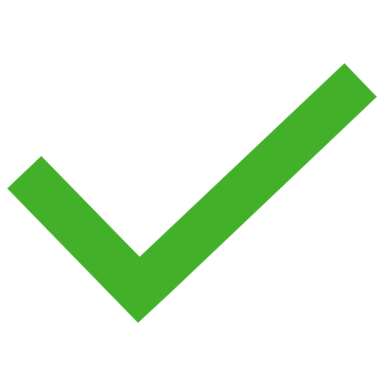** | **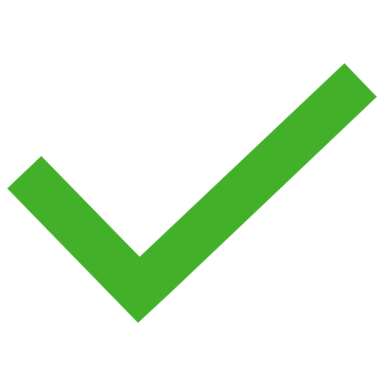** | **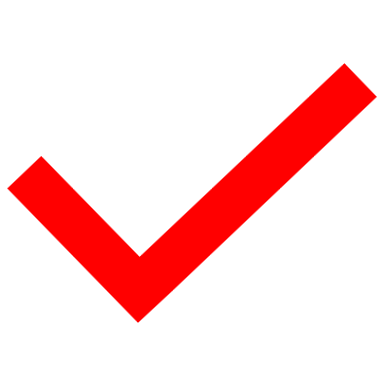** | **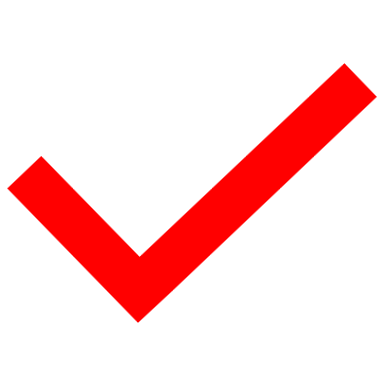** |  |  |  |
| NCT01790126 2020 | Time to PSA progression | **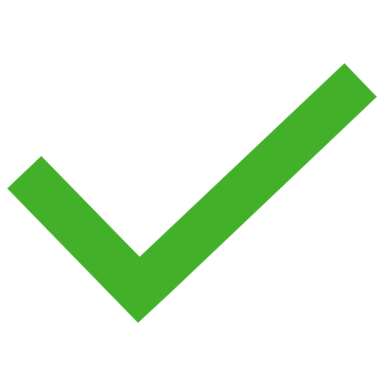** | 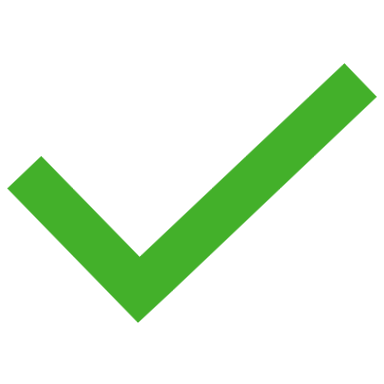 | **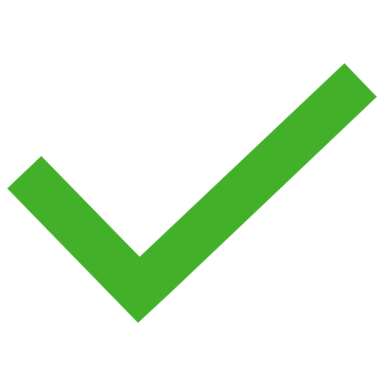** | **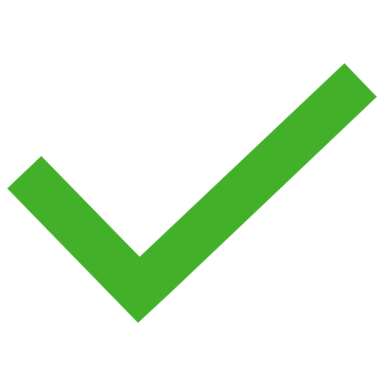** | **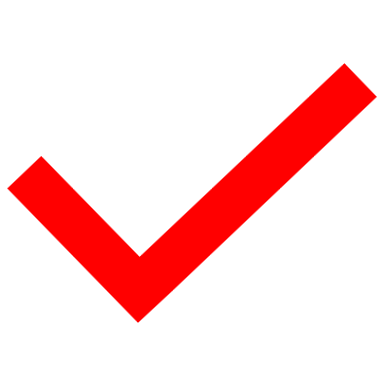** | **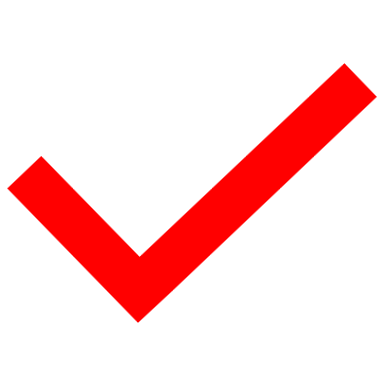** |  |  |  |
| **Safety outcomes** | | | | | | | |  |  |  |
| Autio, 2021 (NR) | Grade ≥3 AEs | **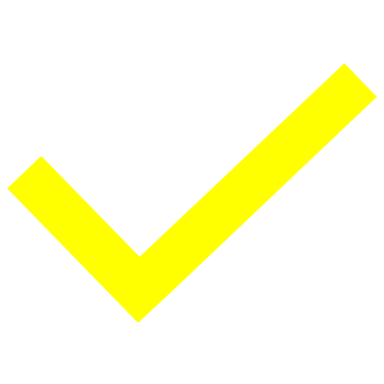** | **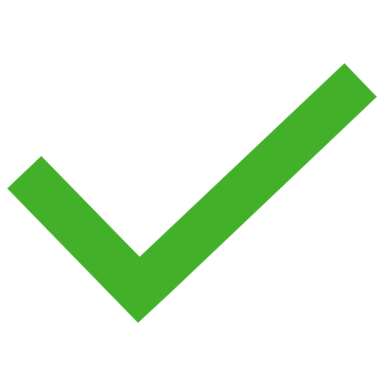** | **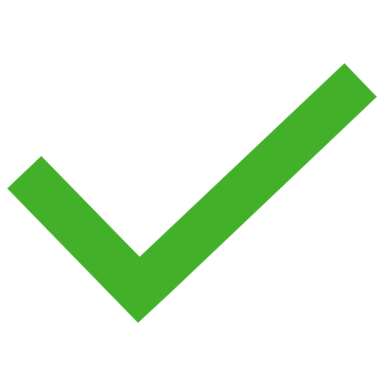** | **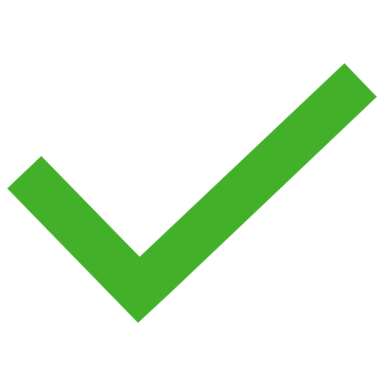** | **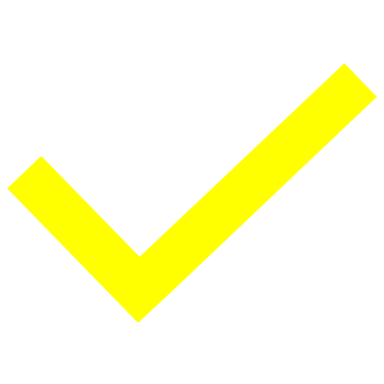** | **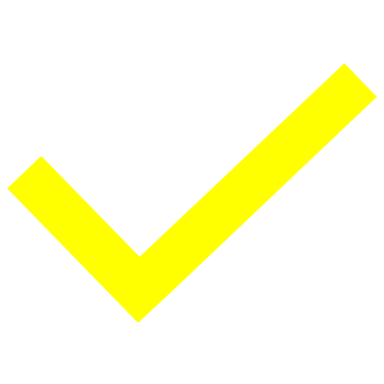** |  |  |  |
| Morris, 2021 (TAX3503) | Grade ≥3 AEs | **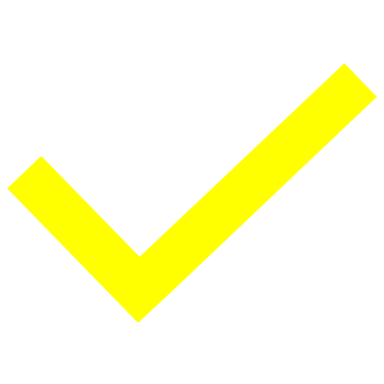** | **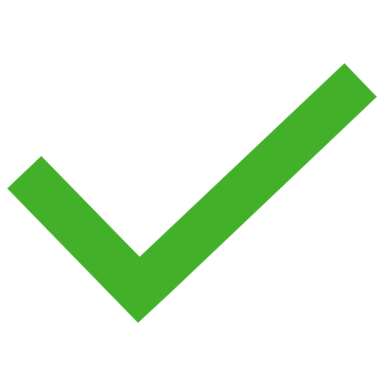** | **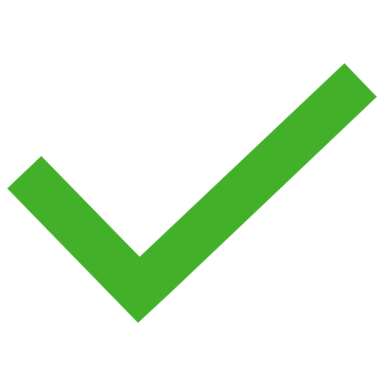** | **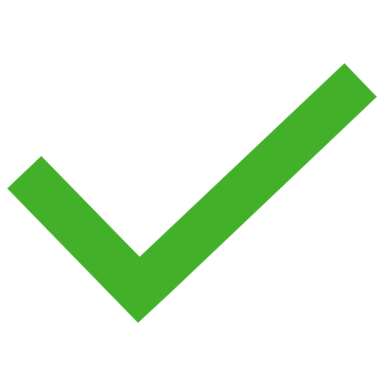** | **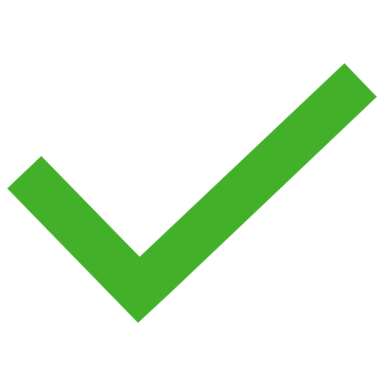** | **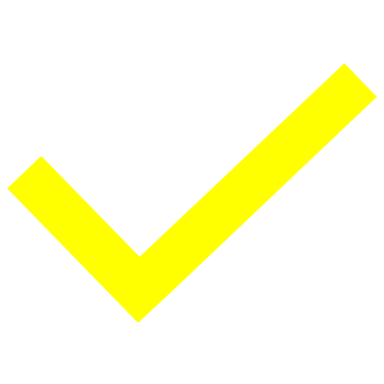** |  |  |  |
| Oudard, 2019 (AOM 03108) | Grade ≥3 AEs | **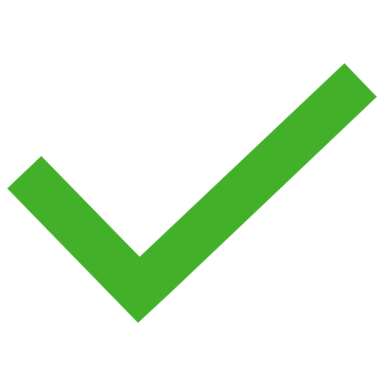** |  |  |  |  |  |  |  |  |
| Spetsieris, 2021 (NR) | Grade ≥3 AEs |  |  |  |  |  |  |  |  |  |

**Abbreviations:** AE: adverse event; NR: not reported; MFS: metastasis-free survival; OS: overall survival; PSA: prostate-specific antigen.

**eTable 5. League table presenting the relative efficacy of enzalutamide (monotherapy and combination therapy) vs comparator treatments as determined from median HR with corresponding 95% CrIs: random-effects models for MFS, OS, time to PSA progression, and time to castration resistance.**

|  | MFS (HR, 95% CrI) | | | | | | | | | | | | | | |
| --- | --- | --- | --- | --- | --- | --- | --- | --- | --- | --- | --- | --- | --- | --- | --- |
|  |  | **Treatments (reference)** | | | | | | | | | | | | | |
|  |  | | | **Enzalutamide** | | | **Enzalutamide + ADT** | | | | **Docetaxel + ADT** | | | **ADT** | |
| Comparators | Enzalutamide | | | - | | | 1.48 (0.04–40.09) | | | | 0.65 (0.01–55.64) | | | 0.68 (0.05–16.37) | |
|  | Enzalutamide + ADT | | | 0.68 (0.02–22.94) | | | - | | | | 0.45 (0.01–39.94) | | | 0.46 (0.03–12.24) | |
|  | Docetaxel + ADT | | | 1.53 (0.02–76.57) | | | 2.22 (0.03–95.56) | | | | - | | | 1.03 (0.05–19.17) | |
|  | ADT | | | 1.48 (0.06–21.94) | | | 2.17 (0.08–29.47) | | | | 0.97 (0.05–19.08) | | | - | |
|  | **OS, HR (95% CrI)** | | | | | | | | | | | | | | |
|  |  | **Treatments (reference)** | | | | | | | | | | | | | |
|  |  | | | **Enzalutamide** | | | **Enzalutamide + ADT** | | | | **Docetaxel + ADT** | | | **ADT** | |
| Comparators | Enzalutamide | | | - | | | 1.31 (0.11–16.35) | | | | 1.10 (0.07–18.17) | | | 0.80 (0.09–7.61) | |
|  | Enzalutamide + ADT | | | 0.76 (0.06–9.43) | | | - | | | | 0.83 (0.06–14.41) | | | 0.60 (0.08–6.16) | |
|  | Docetaxel + ADT | | | 0.91 (0.06–14.35) | | | 1.20 (0.07–17.80) | | | | - | | | 0.72 (0.13–4.26) | |
|  | ADT | | | 1.25 (0.13–11.00) | | | 1.66 (0.16–12.96) | | | | 1.39 (0.23–7.52) | | | - | |
|  | **Time to PSA progression, HR (95% CrI)** | | | | | | | | | | | | | | |
|  |  | **Treatments (reference)** | | | | | | | | | | | | | |
|  |  | | **Enzalutamide** | | | **Enzalutamide + ADT** | | **Docetaxel + ADT** | | **Abiraterone** | | **Abiraterone + ADT** | | | **ADT** |
| Comparators | Enzalutamide | | - | | | 4.68 (1.55–12.82)^a^ | | 0.41 (0.15–1.47) | | 0.17 (0.05–0.77)^a^ | | 0.61 (0.22–2.18) | | | 0.34 (0.15–1.01) |
|  | Enzalutamide + ADT | | 0.21 (0.08–0.65)^a^ | | | - | | 0.09 (0.03–0.36)^a^ | | 0.04 (0.01–0.18)^a^ | | 0.13 (0.04–0.53)^a^ | | | 0.07 (0.03–0.25)^a^ |
|  | Docetaxel + ADT | | 2.42 (0.68–6.69) | | | 11.31 (2.77–34.62)^a^ | | - | | 0.42 (0.15–1.31) | | 1.48 (0.60–3.65) | | | 0.83 (0.44–1.63) |
|  | Abiraterone | | 5.74 (1.30–18.48)^a^ | | | 26.67 (5.50–94.12)^a^ | | 2.37 (0.76–6.86) | | - | | 3.51 (1.35–9.02)^a^ | | | 1.96 (0.78–4.84) |
|  | Abiraterone + ADT | | 1.63 (0.46–4.45) | | | 7.61 (1.88–22.92)^a^ | | 0.67 (0.27–1.66) | | 0.28 (0.11–0.74)^a^ | | - | | | 0.56 (0.30–1.09) |
|  | ADT | | 2.92 (0.99–6.49) | | | 13.59 (3.96–34.94)^a^ | | 1.21 (0.61–2.27) | | 0.51 (0.21–1.28) | | 1.79 (0.92–3.31) | | | - |
|  | **Time to castration resistance** | | | | | | | | | | | | | | |
|  |  | **Treatments (reference)** | | | | | | | | | | | | | |
|  |  | | | | **Enzalutamide + ADT** | | | | **Intermittent ADT** | | | | **ADT** | | |
| Comparators | Enzalutamide + ADT | | | | 1 (1–1) | | | | 0.12 (0.01–9.94) | | | | 0.12 (0.01–13.26) | | |
|  | Intermittent ADT | | | | 8.43 (0.10–171.01) | | | | 1 (1–1) | | | | 0.95 (0.07–25.27) | | |
|  | ADT | | | | 8.30 (0.08–139.80) | | | | 1.06 (0.04–13.57) | | | | 1 (1–1) | | |

**Abbreviations:** ADT: androgen-deprivation therapy; CrI, credible interval; HR: hazard ratio; MFS: metastasis-free survival; OS: overall survival; PSA: prostate-specific antigen.

^a^Statistically significant.

**eTable 6. League table presenting the relative efficacy of enzalutamide (monotherapy and combination therapy) vs comparator treatments as determined from OR with corresponding 95% CrIs: random-effects models for undetectable PSA and grade ≥3 TRAEs.**

|  | Undetectable PSA, OR (95% CrI) | | | | | | | | |
| --- | --- | --- | --- | --- | --- | --- | --- | --- | --- |
|  |  | **Treatments (reference)** | | | | | | | |
|  |  | **Enzalutamide** | **Enzalutamide + ADT** | | **ADT** | | **Abiraterone** | | **Abiraterone + ADT** |
| Comparators | Enzalutamide | - | 0.25 (0.00–241.90) | | 3.59 (0.01–1216.40) | | 1.31 (0.00–2840.24) | | 0.95 (0.00–2508.68) |
|  | Enzalutamide + ADT | 3.97 (0.00–1765.64) | - | | 13.98 (0.01–9572.07) | | 5.05 (0.00–22170.26) | | 3.83 (0.00–21974.50) |
|  | ADT | 0.28 (0.00–109.55) | 0.07 (0.00–89.13) | | - | | 0.36 (0.00–310.62) | | 0.26 (0.00–292.92) |
|  | Abiraterone | 0.76 (0.00–2165.46) | 0.20 (0.00–1863.81) | | 2.76 (0.00–2774.95) | | - | | 0.72 (0.00–1339.65) |
|  | Abiraterone + ADT | 1.06 (0.00–2963.76) | 0.26 (0.00–4446.10) | | 3.85 (0.00–3420.85) | | 1.39 (0.00–1714.47) | | - |
|  | **Grade ≥3 TRAEs, OR (95% CrI)** | | | | | | | | |
|  |  | **Treatments (reference)** | | | | | | | |
|  |  | **Enzalutamide + ADT** | | **ADT** | | **Enzalutamide** | | **Docetaxel + ADT** | |
| Comparators | Enzalutamide + ADT | 1 (1–1) | | 2.26 (0.00–1809.81) | | 1.11 (0.00–1772.53) | | 0.31 (0.00–2544.25) | |
|  | ADT | 0.44 (0.00–490.37) | | 1 (1–1) | | 0.48 (0.00–1226.33) | | 0.13 (0.00–376.80) | |
|  | Enzalutamide | 0.90 (0.00–2030.92) | | 2.07 (0.00–4373.63) | | 1 (1–1) | | 0.28 (0.00–8248.95) | |
|  | Docetaxel + ADT | 3.24 (0.00–43780.53) | | 7.49 (0.00–19824.70) | | 3.55 (0.00–157177.24) | | 1 (1–1) | |

**Abbreviations:** ADT: androgen-deprivation therapy; CrI, credible interval; OR: odds ratio; PSA, prostate-specific antigen; TRAE: treatment-related adverse event.

**eTable 7A. Sensitivity analyses: League table presenting the relative efficacy of enzalutamide (mono and combo) vs comparator treatments in the form of a median HR along with the corresponding 95% credible intervals (CrIs)—fixed-effects model for overall survival**

| **Base case analysis (including EMBARK, Morris et al (2021), and Oudard et al (2019))** | | | | | | | | | |
| --- | --- | --- | --- | --- | --- | --- | --- | --- | --- |
|  |  | **Treatments (reference)** | | | | | | | |
|  |  | **Enzalutamide** | | **Enzalutamide + ADT** | | **Docetaxel + ADT** | | **ADT** | |
| **Comparators** | **Enzalutamide** | - | | 1.32 (1.12–1.56)^a^ | | 1.02 (0.59–1.77) | | 0.78 (0.52–1.16) | |
|  | **Enzalutamide + ADT** | 0.76 (0.64–0.89)^a^ | | - | | 0.77 (0.43–1.37) | | 0.59 (0.38–0.91)^a^ | |
|  | **Docetaxel + ADT** | 0.98 (0.57–1.70) | | 1.30 (0.73–2.30) | | - | | 0.77 (0.53–1.11) | |
|  | **ADT** | 1.28 (0.86–1.91) | | 1.70 (1.09–2.61)^a^ | | 1.31 (0.90–1.90) | | - | |
| **Scenario analysis 1 (including Duchesne et al (2016) in the base case analysis)** | | | | | | | | | |
|  |  | **Treatments (reference)** | | | | | | | |
|  |  | **Enzalutamide** | **Enzalutamide + ADT** | | **Docetaxel + ADT** | | **ADT** | | **Delayed ADT** |
| **Comparators** | **Enzalutamide** | - | 1.32 (1.13–1.55)^a^ | | 1.02 (0.59–1.76) | | 0.78 (0.52–1.16) | | 0.45 (0.21–0.98)^a^ |
|  | **Enzalutamide + ADT** | 0.76 (0.64–0.89)^a^ | - | | 0.77 (0.44–1.36) | | 0.59 (0.38–0.91)^a^ | | 0.34 (0.16–0.75)^a^ |
|  | **Docetaxel + ADT** | 0.98 (0.57–1.69) | 1.30 (0.74–2.29) | | - | | 0.76 (0.53–1.10) | | 0.44 (0.21–0.94)^a^ |
|  | **ADT** | 1.29 (0.86–1.92) | 1.70 (1.10–2.62)^a^ | | 1.31 (0.91–1.90) | | - | | 0.58 (0.30–1.11) |
|  | **Delayed ADT** | 2.22 (1.02–4.79)^a^ | 2.94 (1.33–6.42)^a^ | | 2.26 (1.07–4.82)^a^ | | 1.73 (0.90–3.33) | | - |
| **Scenario analysis 2 (excluding Morris et al (2021) from the base case analysis)** | | | | | | | | | |
|  |  | **Treatments (reference)** | | | | | | | |
|  |  | **Enzalutamide** | | **Enzalutamide + ADT** | | **Docetaxel + ADT** | | **ADT** | |
| **Comparators** | **Enzalutamide** | - | | 1.32 (1.12–1.56)^a^ | | 0.90 (0.51–1.61) | | 0.78 (0.52–1.17) | |
|  | **Enzalutamide + ADT** | 0.76 (0.64–0.89)^a^ | | - | | 0.69 (0.37–1.25) | | 0.59 (0.38–0.91)^a^ | |
|  | **Docetaxel + ADT** | 1.11 (0.62–1.98) | | 1.46 (0.80–2.67) | | - | | 0.86 (0.56–1.31) | |
|  | **ADT** | 1.28 (0.86–1.92) | | 1.70 (1.10–2.64)^a^ | | 1.16 (0.76–1.78) | | - | |
| **Scenario analysis 3 (including Duchesne et al (2016) and excluding Morris et al (2021) from the base case analysis)** | | | | | | | | | |
|  |  | **Treatments (reference)** | | | | | | | |
|  |  | **Enzalutamide** | **Enzalutamide + ADT** | | **Docetaxel + ADT** | | **ADT** | | **Delayed ADT** |
| **Comparators** | **Enzalutamide** | - | 1.32 (1.12–1.55)^a^ | | 0.91 (0.51–1.64) | | 0.78 (0.52–1.17) | | 0.46 (0.21–0.99)^a^ |
|  | **Enzalutamide + ADT** | 0.76 (0.64–0.89)^a^ | - | | 0.69 (0.38–1.27) | | 0.59 (0.38–0.91)^a^ | | 0.35 (0.16–0.76)^a^ |
|  | **Docetaxel + ADT** | 1.10 (0.61–1.96) | 1.45 (0.79–2.66) | | - | | 0.86 (0.56–1.31) | | 0.50 (0.23–1.09) |
|  | **ADT** | 1.28 (0.85–1.91) | 1.69 (1.09–2.62)^a^ | | 1.17 (0.76–1.78) | | - | | 0.58 (0.30–1.12) |
|  | **Delayed ADT** | 2.19 (1.01–4.76)^a^ | 2.90 (1.32–6.41)^a^ | | 2.00 (0.92–4.39) | | 1.71 (0.89–3.33) | | - |
| **Scenario analysis 4 (considering ADT regimen in EMBARK is intermittent and including Crook et al (2012))** | | | | | | | | | |
|  |  | **Treatment (reference)** | | | | | | | |
|  |  | **Enzalutamide** | **Enzalutamide + Intermittent ADT** | | **Docetaxel + ADT** | | **Intermittent ADT** | | **ADT** |
| **Comparators** | **Enzalutamide** | - | 1.32 (1.12–1.56)^a^ | | 0.98 (0.60–1.59) | | 0.78 (0.52–1.17) | | 0.80 (0.52–1.25) |
|  | **Enzalutamide + Intermittent ADT** | 0.76 (0.64–0.89)^a^ | - | | 0.74 (0.44–1.24) | | 0.59 (0.38–0.91)^a^ | | 0.61 (0.38–0.97)^a^ |
|  | **Docetaxel + ADT** | 1.02 (0.63–1.65) | 1.35 (0.81–2.25) | | - | | 0.80 (0.61–1.05) | | 0.82 (0.67–1.01) |
|  | **Intermittent ADT** | 1.28 (0.86–1.92) | 1.69 (1.10–2.61)^a^ | | 1.25 (0.96–1.64) | | - | | 1.03 (0.86–1.23) |
|  | **ADT** | 1.24 (0.80–1.92) | 1.65 (1.03–2.62)^a^ | | 1.22 (0.99–1.49) | | 0.97 (0.81–1.16) | | - |

^a^Statistically significant.

**eTable 7B. Sensitivity analyses: League table presenting the relative efficacy of enzalutamide (mono and combo) vs comparator treatments in the form of a median HR along with the corresponding 95% credible intervals (CrIs)—fixed effect model for time to PSA progression**

| **Base case analysis (including EMBARK, Autio et al (2021), Morris et al (2021), Oudard et al (2019), and Spetsieris et al (2021))** | | |
| --- | --- | --- |
|  |  | **Treatments (reference)** |

|  |  | **Enzalutamide** | **Enzalutamide + ADT** | **Docetaxel + ADT** | **Abiraterone** | **Abiraterone + ADT** | **ADT** |
| --- | --- | --- | --- | --- | --- | --- | --- |
| **Comparators** | **Enzalutamide** | - | 4.85 (2.62–8.98)^a^ | 0.40 (0.26–0.62)^a^ | 0.17 (0.09–0.32)^a^ | 0.60 (0.39–0.93)^a^ | 0.33 (0.23–0.49)^a^ |
|  | **Enzalutamide + ADT** | 0.21 (0.11–0.38)^a^ | - | 0.08 (0.04–0.18)^a^ | 0.04 (0.01–0.09)^a^ | 0.12 (0.06–0.26)^a^ | 0.07 (0.03–0.14)^a^ |
|  | **Docetaxel + ADT** | 2.48 (1.62–3.79)^a^ | 12.00 (5.66–25.67)^a^ | - | 0.43 (0.25–0.72)^a^ | 1.49 (1.14–1.95)^a^ | 0.82 (0.68–0.99)^a^ |
|  | **Abiraterone** | 5.81 (3.12–10.91)^a^ | 28.13 (11.68–68.21)^a^ | 2.35 (1.39–3.97)^a^ | - | 3.50 (2.08–5.94)^a^ | 1.93 (1.19–3.15)^a^ |
|  | **Abiraterone + ADT** | 1.66 (1.08–2.55)^a^ | 8.05 (3.80–17.28)^a^ | 0.67 (0.51–0.88)^a^ | 0.29 (0.17–0.48)^a^ | - | 0.55 (0.46–0.67)^a^ |
|  | **ADT** | 3.01 (2.05–4.43)^a^ | 14.57 (7.07–30.34)^a^ | 1.21 (1.01–1.47)^a^ | 0.52 (0.32–0.84)^a^ | 1.81 (1.50–2.19)^a^ | - |

| **Scenario analysis 1 (excluding Morris et al (2021))** | | |
| --- | --- | --- |
|  |  | **Treatments (reference)** |

|  |  | **Enzalutamide** | **Enzalutamide + ADT** | **Docetaxel + ADT** | **Abiraterone** | **Abiraterone + ADT** | **ADT** |
| --- | --- | --- | --- | --- | --- | --- | --- |
| **Comparators** | **Enzalutamide** | - | 4.86 (2.59–8.97)^a^ | 0.39 (0.24–0.64)^a^ | 0.17 (0.09–0.32)^a^ | 0.60 (0.39–0.92)^a^ | 0.33 (0.23–0.48)^a^ |
|  | **Enzalutamide + ADT** | 0.21 (0.11–0.39)^a^ | - | 0.08 (0.04–0.18)^a^ | 0.04 (0.01–0.09)^a^ | 0.12 (0.06–0.26)^a^ | 0.07 (0.03–0.14)^a^ |
|  | **Docetaxel + ADT** | 2.57 (1.56–4.20)^a^ | 12.44 (5.62–27.61)^a^ | - | 0.44 (0.24–0.79)^a^ | 1.54 (1.06–2.23)^a^ | 0.85 (0.62–1.17) |
|  | **Abiraterone** | 5.82 (3.14–10.95)^a^ | 28.28 (11.66–68.58)^a^ | 2.27 (1.27–4.09)^a^ | - | 3.50 (2.06–5.96)^a^ | 1.93 (1.18–3.17)^a^ |
|  | **Abiraterone + ADT** | 1.67 (1.09–2.58)^a^ | 8.08 (3.80–17.24)^a^ | 0.65 (0.45–0.94)^a^ | 0.29 (0.17–0.49)^a^ | - | 0.55 (0.46–0.67)^a^ |
|  | **ADT** | 3.02 (2.07–4.44)^a^ | 14.62 (7.01–30.32)^a^ | 1.18 (0.86–1.61) | 0.52 (0.32–0.85)^a^ | 1.81 (1.50–2.19)^a^ | - |

| **Scenario analysis 2 (excluding Spetsieris et al (2021))** | | |
| --- | --- | --- |
|  |  | **Treatments (reference)** |

|  |  | **Enzalutamide** | **Enzalutamide + ADT** | **Docetaxel + ADT** | **Abiraterone** | **Abiraterone + ADT** | **ADT** |
| --- | --- | --- | --- | --- | --- | --- | --- |
| **Comparators** | **Enzalutamide** | - | 4.85 (2.61–8.94)^a^ | 0.40 (0.26–0.63)^a^ | 0.17 (0.09–0.32)^a^ | 0.66 (0.42–1.04) | 0.33 (0.23–0.49)^a^ |
|  | **Enzalutamide + ADT** | 0.21 (0.11–0.38)^a^ | - | 0.08 (0.04–0.18)^a^ | 0.04 (0.01–0.09)^a^ | 0.14 (0.06–0.29)^a^ | 0.07 (0.03–0.14)^a^ |
|  | **Docetaxel + ADT** | 2.48 (1.60–3.80)^a^ | 12.03 (5.67–25.55)^a^ | - | 0.43 (0.25–0.72)^a^ | 1.63 (1.20–2.21)^a^ | 0.82 (0.68–0.99)^a^ |
|  | **Abiraterone** | 5.82 (3.09–10.87)^a^ | 28.09 (11.62–67.81)^a^ | 2.35 (1.38–3.99)^a^ | - | 3.82 (2.20–6.59)^a^ | 1.93 (1.18–3.16)^a^ |
|  | **Abiraterone + ADT** | 1.52 (0.96–2.40) | 7.37 (3.44–15.92)^a^ | 0.61 (0.45–0.83)^a^ | 0.26 (0.15–0.46)^a^ | - | 0.51 (0.40–0.64)^a^ |
|  | **ADT** | 3.02 (2.03–4.43)^a^ | 14.58 (7.03–30.29)^a^ | 1.21 (1.01–1.47)^a^ | 0.52 (0.32–0.85)^a^ | 1.98 (1.56–2.51)^a^ | - |

| **Scenario analysis 3 (including Aggarwal et al (2023))*** | | | | | | | | | | | | | | |
| --- | --- | --- | --- | --- | --- | --- | --- | --- | --- | --- | --- | --- | --- | --- |
|  |  | **Treatments (reference)** | | | | | | | | | | | | |
|  |  | **Enzalutamide** | **Enzalutamide + ADT** | | **Docetaxel + ADT** | | **Abiraterone** | | **Abiraterone + ADT** | **Apalutamide + ADT** | | **Apalutamide + Abiraterone + ADT** | | **ADT** |
| **Comparators** | **Enzalutamide** | - | 4.87  (2.62–9.03)^a^ | | 0.40  (0.26–0.61)^a^ | | 0.37  (0.22–0.61)^a^ | | 0.65  (0.40–1.03) | 0.64  (0.36–1.10) | | 0.69  (0.39–1.20) | | 0.33  (0.22–0.48)^a^ |
|  | **Enzalutamide + ADT** | 0.21  (0.11–0.38)^a^ | - | | 0.08  (0.04–0.17)^a^ | | 0.08  (0.03–0.17)^a^ | | 0.13  (0.06–0.29)^a^ | 0.13  (0.06–0.30)^a^ | | 0.14  (0.06–0.33)^a^ | | 0.07  (0.03–0.14)^a^ |
|  | **Docetaxel + ADT** | 2.49  (1.63–3.85)^a^ | 12.10  (5.73–25.93)^a^ | | - | | 0.92  (0.64–1.32) | | 1.60  (1.16–2.22)^a^ | 1.58  (1.02–2.46)^a^ | | 1.71  (1.10–2.67)^a^ | | 0.82  (0.68–0.99)^a^ |
|  | **Abiraterone** | 2.70  (1.65–4.47)^a^ | 13.14  (5.94–29.18)^a^ | | 1.09  (0.76–1.56) | | - | | 1.74  (1.39–2.17)^a^ | 1.72  (1.04–2.83)^a^ | | 1.86  (1.12–3.08)^a^ | | 0.90  (0.66–1.22) |
|  | **Abiraterone + ADT** | 1.55  (0.97–2.51) | 7.57  (3.46–16.49)^a^ | | 0.62  (0.45–0.86)^a^ | | 0.58  (0.46–0.72)^a^ | | - | 0.99  (0.61–1.59) | | 1.07  (0.66–1.73) | | 0.51  (0.39–0.67)^a^ |
|  | **Apalutamide + ADT** | 1.57  (0.91–2.75) | 7.65  (3.33–17.57)^a^ | | 0.63  (0.41–0.98)^a^ | | 0.58  (0.35–0.96)^a^ | | 1.01  (0.63–1.64) | - | | 1.08  (1.02–1.15)^a^ | | 0.52  (0.35–0.78)^a^ |
|  | **Apalutamide + Abiraterone + ADT** | 1.45  (0.83–2.55) | 7.06  (3.08–16.26)^a^ | | 0.58  (0.37–0.91)^a^ | | 0.54  (0.32–0.89)^a^ | | 0.93  (0.58–1.52) | 0.92  (0.87–0.98) | | - | | 0.48  (0.32–0.72) |
|  | **ADT** | 3.02  (2.06–4.47) | 14.69  (7.09–30.56)^a^ | | 1.21  (1.01–1.46) | | 1.12  (0.82–1.52) | | 1.94  (1.49–2.55)^a^ | 1.92  (1.29–2.86)^a^ | | 2.08  (1.39–3.11)^a^ | | - |
| **Scenario analysis 4 (excluding Morris et al (2021) and Spetsieris et al (2021))** | | | | | | | | | | | | | | |
|  |  | **Treatments (reference)** | | | | | | | | | | | | |
|  |  | **Enzalutamide** | | **Enzalutamide + ADT** | | **Docetaxel + ADT** | | **Abiraterone** | | | **Abiraterone + ADT** | | **ADT** | |
| **Comparators** | **Enzalutamide** | - | | 4.87 (2.63–8.98)^a^ | | 0.39 (0.24–0.64)^a^ | | 0.64 (0.34–1.19) | | | 1.27 (0.65–2.45) | | 0.33 (0.23–0.48)^a^ | |
|  | **Enzalutamide + ADT** | 0.21 (0.11–0.38)^a^ | | - | | 0.08 (0.04–0.18)^a^ | | 0.13 (0.06–0.31) | | | 0.26 (0.11–0.64)^a^ | | 0.07 (0.03–0.14)^a^ | |
|  | **Docetaxel + ADT** | 2.56 (1.57–4.22)^a^ | | 12.49 (5.63–27.79)^a^ | | - | | 1.65 (0.92–2.93) | | | 3.26 (1.74–6.10)^a^ | | 0.85 (0.62–1.16) | |
|  | **Abiraterone** | 1.56 (0.84–2.91) | | 7.58 (3.18–18.17)^a^ | | 0.61 (0.34–1.08) | | - | | | 1.98 (1.56–2.52)^a^ | | 0.51 (0.32–0.84)^a^ | |
|  | **Abiraterone + ADT** | 0.79 (0.41–1.54) | | 3.82 (1.56–9.49)^a^ | | 0.31 (0.16–0.57)^a^ | | 0.51 (0.40–0.64)^a^ | | | - | | 0.26 (0.15–0.45)^a^ | |
|  | **ADT** | 3.02 (2.07–4.44)^a^ | | 14.71 (7.14–30.27)^a^ | | 1.18 (0.86–1.60) | | 1.94 (1.19–3.15)^a^ | | | 3.83 (2.23–6.60)^a^ | | - | |

^a^Statistically significant.

*Time to PSA progression was called “PSA progression-free survival” in the study by Aggarwal et al.^33^ However, as the definition of this outcome was similar to how we defined time to PSA progression, these outcomes were assumed to be equivalent for the purpose of this sensitivity analysis.

**eFigure 1. Network of evidence diagrams**

**A. Network of evidence for OS**

**B. Network of evidence for MFS**

**C. Network of evidence for time to PSA progression**

**D. Network of evidence for time to castration resistance**

**E. Network of evidence for undetectable PSA**

**F. Network of evidence for grade ≥3 TRAEs**

**Abbreviations:** ABI: abiraterone; ADT: androgen-deprivation therapy; DOC: docetaxel; ENZ: enzalutamide; MFS: metastasis-free survival; OS: overall survival; PSA: prostate-specific antigen; TRAE: treatment-related adverse event.

**Supplementary Figure 2. Schoenfeld residuals and log-log hazard plots of EMBARK IPD for the assessment of proportional hazards in MFS (A, B), OS (C, D), time to PSA progression (E, F), and time to castration resistance (G, H**

)

**Abbreviations:** IPD: individual patient data; MFS: metastasis-free survival; OS: overall survival; PSA: prostate-specific antigen.

**Supplementary Figure 3A. Forest plot representing the relative efficacy of enzalutamide + ADT against other active treatments as produced by FE model: metastasis-free survival**

**Supplementary Figure 3B. Forest plot representing the relative efficacy of enzalutamide monotherapy against other active treatments as produced by FE model: metastasis-free survival**

**Supplementary Figure 3C. Forest plot representing the relative efficacy of enzalutamide + ADT against other active treatments as produced by FE model: time to PSA progression**

**Supplementary Figure 3D. Forest plot representing the relative efficacy of enzalutamide monotherapy against other active treatments as produced by FE model: time to PSA progression**

**Supplementary Figure 3E. Forest plot representing the relative efficacy of enzalutamide + ADT against other active treatments as produced by FE model: time to castration resistance**

**Supplementary Figure 3F. Forest plot representing the relative efficacy of enzalutamide + ADT against other active treatments as produced by FE model: proportion of patients with undetectable PSA at 36 (±4) weeks of treatment**

**Supplementary Figure 3G. Forest plot representing the relative efficacy of enzalutamide monotherapy against other active treatments as produced by FE model: proportion of patients with undetectable PSA at 36 (±4) weeks of treatment**

**Supplementary Figure 3H. Forest plot representing the relative efficacy of enzalutamide + ADT against other active treatments as produced by FE model: grade ≥3 TRAEs**

**Supplementary Figure 3I. Forest plot representing the relative efficacy of enzalutamide monotherapy against other active treatments as produced by FE model: grade ≥3 TRAEs**

**Abbreviations:** ADT: androgen-deprivation therapy; CrI: credible interval; PSA: prostate-specific antigen; TRAE: treatment-related adverse event.
